# scGIST: gene panel design for spatial transcriptomics with prioritized gene sets

**DOI:** 10.1186/s13059-024-03185-y

**Published:** 2024-02-26

**Authors:** Mashrur Ahmed Yafi, Md. Hasibul Husain Hisham, Francisco Grisanti, James F. Martin, Atif Rahman, Md. Abul Hassan Samee

**Affiliations:** 1 Department of Computer Science and Engineering, Bangladesh University of Engineering and Technology, Dhaka, 1205 Bangladesh; 2 Department of Integrative Physiology, Baylor College of Medicine, Houston, 77030 TX USA; 3 Cardiomyocyte Renewal Laboratory, Texas Heart Institute, Houston, 77030 TX USA

## Abstract

A critical challenge of single-cell spatial transcriptomics (sc-ST) technologies is their panel size. Being based on fluorescence in situ hybridization, they are typically limited to panels of about a thousand genes. This constrains researchers to build panels from only the marker genes of different cell types and forgo other genes of interest, e.g., genes encoding ligand-receptor complexes or those in specific pathways. We propose scGIST, a constrained feature selection tool that designs sc-ST panels prioritizing user-specified genes without compromising cell type detection accuracy. We demonstrate scGIST’s efficacy in diverse use cases, highlighting it as a valuable addition to sc-ST’s algorithmic toolbox.

**Supplementary Information:**

The online version contains supplementary material available at 10.1186/s13059-024-03185-y.

## Introduction

Spatial transcriptomics (ST) is an emerging suite of technologies that quantify spatially resolved gene expression from intact tissue sections [1–4]. Commercially available ST technologies fall into two types [1–4]. The spot-based ST (spot-ST) technologies quantify gene expression in spatially resolved spots where each spot captures about 10 cells. These technologies are sequencing-based and, in principle, capture the transcriptome at each spot. However, the lack of single-cell resolution makes spot-ST data challenging to use in single-cell focused studies, e.g., to analyze the spatial variation in ligand-receptor usage of a given cell-type [5].

In contrast to spot-ST, the alternative single-cell ST (sc-ST) technologies capture data from individual cells while recording their locations in the tissue section [3]. However, being fluorescence in situ hybridization (FISH)-based, each sc-ST dataset is limited to a pre-determined panel of 10–250 genes [6–12]. Commercially available pipelines implementing technologies like MERFISH (multiplexed error-robust FISH), EEL FISH (Enhanced ELectric FISH), and SMI (spatial molecular imager) have started offering panels of about a thousand genes [13–16] and, in the academic setting, seqFISH+ (sequential FISH) and RNA SPOTs (RNA sequential probing of targets) have successfully used about 10,000 genes [17, 18]. Yet, when one wishes elaborate cataloging of cell states (or cell subtypes) and needs to profile additional genes in scenarios like those discussed below, panel sizes remain a bottleneck. This constraint on panel size often limits the potential use of sc-ST data. One primarily prioritizes the marker genes of different cell types in the panel so that the dataset reveals a spatial map of those cell types. However, the panel forgoes other genes of interest relevant to the biological question, e.g., receptors-ligands, genes in a specific pathway, or genes dysregulated in disease conditions. Thus, there is a critical need for algorithms to design sc-ST gene panels that (a) accommodate genes of interest beyond cell type markers while (b) accurately detecting different cell types and (c) remaining within the panel’s size limit. Importantly, these algorithms should be designed to deal with constraints that biologists face in their practical applications. For example, when a biologist wishes to set differential priorities to the genes of interest, the algorithm should maximize the inclusion of higher priority genes. Similarly, when a biologist wants to include protein complexes (such as ligand-receptor complexes) in the panel, the algorithm should aim at including all genes encoding the proteins in a complex. To address these versatile needs while ensuring cell type detection accuracy, here we present scGIST (single-cell Gene-panel Inference for Spatial Transcriptomics).

scGIST is a deep neural network with a custom loss function that casts sc-ST panel design as a constrained feature selection problem [19]. Given a single-cell (sc) RNA-seq dataset annotated for cell types, scGIST learns to classify the individual cells given their gene expression values. Notably, its custom loss function aims at maximizing both cell type classification accuracy and the number of genes included from a given gene set of interest while staying within the panel’s size constraint. Thanks to this loss function, scGIST can cater to a wide range of applications and enable gene panel design to address specific research questions. For example, as an input to the model, a biologist can specify differential priorities for genes in the given gene set. Such scenarios may arise when they wish to assign higher priorities to genes that are easy to target using FISH probes or lower priorities to genes that are expressed above a threshold level (to avoid optical crowding). In such cases, scGIST will try to maximize the number of highest priority genes in the panel without compromising cell type prediction accuracy to a great extent. scGIST also allows users to specify a list of genes of interest or protein complexes, such as ligand-receptor complexes. For every complex, the model will aim at selecting the complete set of genes encoding the proteins in that complex.

We demonstrate scGIST’s efficacy and versatility by benchmarking it on four datasets comprising 2700–20,000 cells and 8–23 cell types, and through a number of use cases. We are unaware of other methods with such generic applicability as scGIST. Three recent sc-ST marker selection methods, namely scGeneFit [20], geneBasis [21], and SMaSH [22], can be used to identify marker genes for a given panel size. scGeneFit [20] selects marker genes given scRNA-seq data and cell labels using linear optimization techniques. geneBasis [21] designs panels by greedily adding a new gene that captures the maximum Minkowski distance between the true manifold and the current manifold. It allows a priori addition of genes of interest to the panel but, unlike scGIST, cannot trade-off their inclusion with accuracy. SMaSH [22] is a machine learning framework which learns a classifier for cell types and then greedily selects markers using various criteria.

We first compare scGIST with these three methods [20–22] in a setting with no prioritized genes. Our benchmarking demonstrates that scGIST consistently outperforms the alternative methods and the margins are remarkable for small panel sizes ($<50$). Next, we show that scGIST can include other genes or complexes of interest such as genes that are easy to target, genes in specific pathways, and receptor-ligand complexes in the panel without sacrificing cell type prediction accuracy substantially. Overall, with its high accuracy and versatile applicability, scGIST addresses a critical need in current sc-ST methods.

## Results

### A deep learning model for marker gene selection

**Fig. 1:**
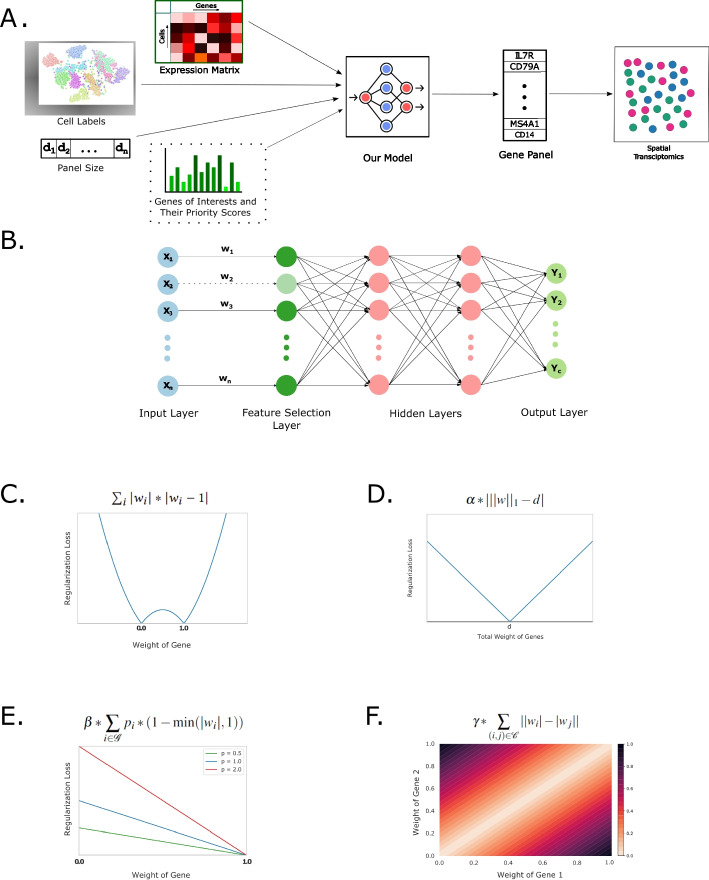
Overview of scGIST. **A** scGIST takes as input expression values from an scRNA-seq experiment, labels for the cells, a target gene panel size, and optionally a list of genes of interest and their priority scores. It uses a deep learning model to learn a set of genes to predict cell types which can be used as a gene panel for single-cell spatial transcriptomics assays. **B** The neural network model of scGIST consists of a one-to-one feature selection layer connected to the input layer. This is then followed by two fully connected layers and finally an output layer. The model has a custom loss function to **C** drive weights in the feature selection layer towards 0 or 1, **D** select the number of genes equal to the panel size (*d*), **E** prioritize genes of interest according to user provided priority scores ($p_i$), and **F** select both or none of the genes from specified complexes

We solve the gene panel selection problem by formulating it as a feature selection problem for multi-class classification in neural networks [19]. Our method, illustrated in Fig. 1A, takes as input gene expression values from an scRNA-seq experiment, labels for the cells, and a target gene panel size. The cell labels may represent cell states or cell types. If the labels are not available, they may be obtained by clustering, or through other approaches [23]. It then generates as output a gene panel that can accurately distinguish among cell types. These genes can subsequently be used as marker genes for technologies such as spatial transcriptomics.

At the core of our method, there is a deep learning model for multi-class classification with a custom loss function for feature selection. The inputs to this neural network are the log-normalized counts of the scRNA-seq data and the outputs are the probabilities of each type of cell. The neural network, shown in Fig. 1B, contains a one-to-one linear layer (feature selection layer) between the input layer and the first fully connected layer, which is then followed by 2 fully connected layers. Finally, there is an output layer that predicts the labels of the cells. To perform feature selection, we use a custom loss function [19] which includes the following components (see the “Methods” section for details):

- A regularization term in the one-to-one linear layer that drives the weights of each gene towards either 0 or 1 (Fig. 1C). This term promotes the sparsity of the weights. Additionally, a regularization term corresponding to all other layers ensures that no small weight in the one-to-one layer is amplified by any of the subsequent layers.
- A penalty term that comes into effect if the sum of the weights of the one-to-one layer is not equal to the target panel size *d* (Fig. 1D). Since these weights are close to zero or one, this sum represents the number of features taken. This term can also optionally be set to penalize only if the sum of the weights exceeds *d* (Additional file 1: Supplementary Note 3.1).

The model is then trained using the provided scRNA-seq expression values and the cell labels, and the genes corresponding to the *d* highest weights in the first layer are selected as the gene panel.

### Regularization for inclusion of other genes and complexes of interest

In addition to marker gene selection, scGIST allows users to provide as an option a list of genes of interest and their priority scores. These genes may represent known marker genes, easy-to-target genes, and other genes and complexes of interest. The method places a greater emphasis on inclusion of the user specified genes based on their priority scores. This is done as follows (details in the “Methods” section):

- scGIST may be provided with lists of genes of interest to be included in the panel along with their priority scores optionally. For each user specified gene, a penalty proportional to its priority score is added if it is not selected in the panel (Fig. 1E).
- Users may also list receptor-ligand or other complexes for inclusion in the gene panel. For each pair of genes in the complexes, a penalty term proportional to the difference of their weights in the one-to-one layer is added to the loss function (Fig. 1F). As a result, the inclusion of only a subset of the genes in a complex will result in an additional cost.

To assess the performance of scGIST, we use it to analyze four real datasets—peripheral blood mononuclear cells (PBMC 3k) (https://support.10xgenomics.com/single-cell-gene-expression/datasets/1.1.0/pbmc3k), Head and Neck Cancer [24], Tabula Sapiens [25], and Mouse Endoderm [26]. First, we present a comparison of its performance with those of the existing gene panel selection tools scGeneFit, geneBasis, and SMaSH and subsequently analyze its effectiveness in including user provided genes and complexes of interest.

### Gene panel selection for cell type identification

**Fig. 2:**
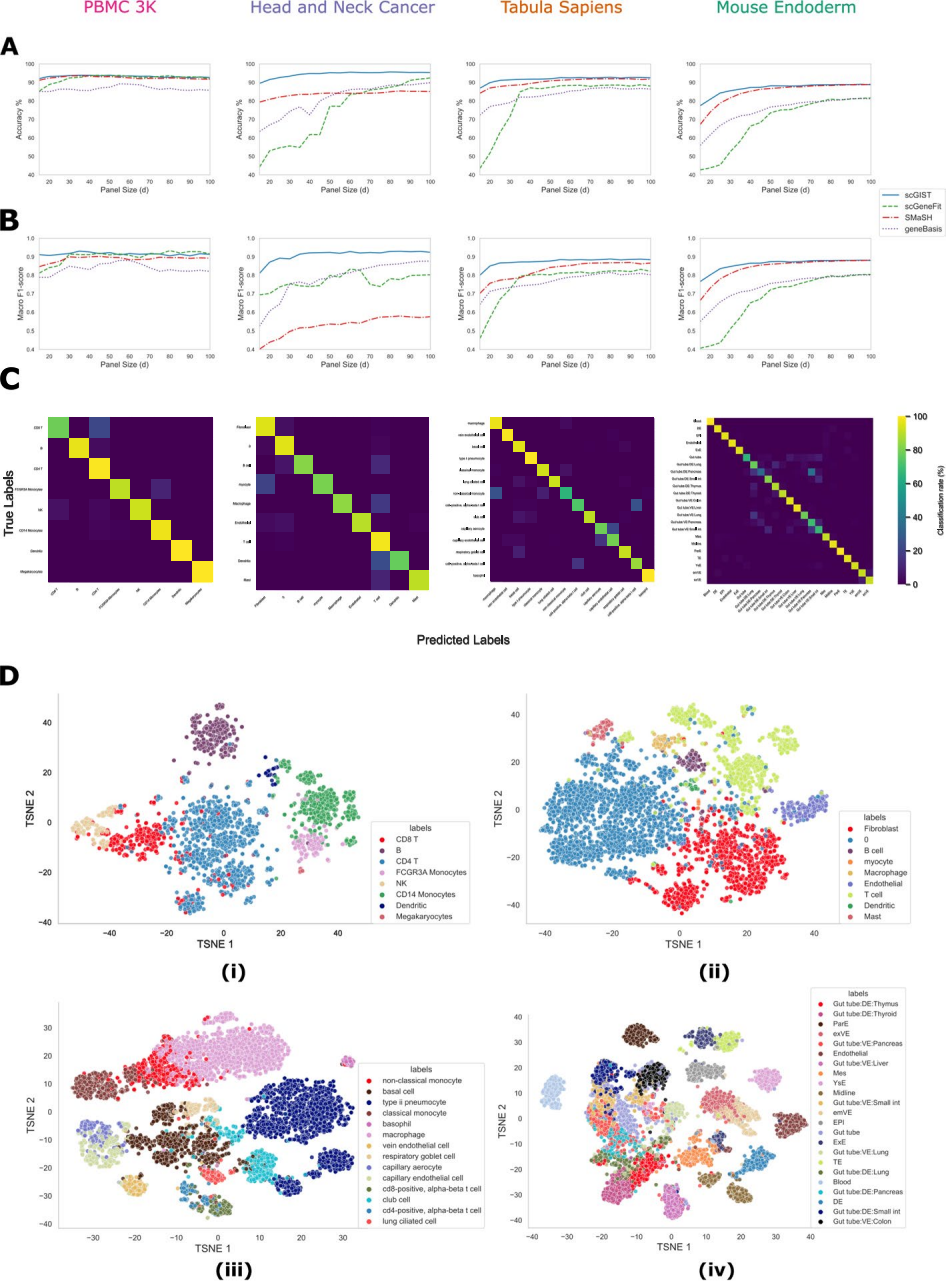
Performance of various methods on four different datasets. **A** Accuracies for variable panel sizes. **B** Macro F1-scores for variable panel sizes. **C** Confusion matrices for scGIST for panel size = 60. **D** t-SNE plots using the gene panel of size 60 selected by scGIST for (i) PBMC 3K, (ii) Head and Neck Cancer, (iii) Tabula Sapiens, and (iv) Mouse Endoderm datasets

We ran scGIST as well as scGeneFit, geneBasis, and SMaSH on the four aforementioned datasets for varying panel sizes and calculated their accuracy (Fig. 2A and Additional file 1: Tables S1, S3, S5, and S7). The values for scGIST are averages over five runs. Additional file 1: Supplementary Note 3.3 shows the values for each run and indicates that the results are stable across runs. We observe that our method performs as well as the state-of-the-art methods in the PBMC 3K dataset and outperforms all methods in the other three datasets. scGIST often outperforms other methods for marker selection substantially, which is especially noticeable for small panel sizes. As an example, for the Head and Neck Cancer dataset, scGIST has an accuracy of 95.31% at panel size of 50 whereas scGeneFit, geneBasis, and SMaSH have accuracies of 77.18%, 82.67%, and 84.25% respectively. For smaller panel sizes, the differences are even more pronounced.

Macro F1 scores (Additional file 1: Supplementary Note 3.2) were also computed for the methods for different panel sizes as accuracy can be misleading when there is class imbalance. We find that scGIST outperforms other methods in terms of macro F1 score as well (Fig. 2B and Additional file 1: Tables S2,S4,S6,S8). It is worth noting that although SMaSH has more than 80% accuracy in the Head and Neck Cancer dataset, it has a low macro F1-score in that dataset. For example, it has a macro F1-score of 0.58 despite having an accuracy of 85.06% at panel size of 100. The high macro F1 scores of our method are due to its accuracy across all cell types which is illustrated in the confusion matrices in Fig. 2C.

We also created t-SNE plots [27] for visualization of the classification into various cell types (Fig. 2D). For each of the four datasets, 60 marker genes were first selected by our method and then t-SNE plots were created using those genes. It can be observed from the plots that the genes selected by our method can largely separate the different cell types in all datasets. It is worth noting that all four methods perform poorly in distinguishing various gut tube cell types in the Mouse Endoderm dataset compared to all other cell types in all datasets.

### Performance on rare cell types

As indicated by the macro F1-scores, scGIST performs well on rare cell types. Figure 2C and Additional file 1: Figs. S2–S5 show the cell type confusion matrices of scGIST as well as scGeneFit, geneBasis, and SMaSH for the four datasets for panel size of 60. Cell type abundances are shown in ordered bar plots in Additional file 1: Fig. S1. We observe that scGIST, scGeneFit, and geneBasis show consistent performance across all cell types with scGIST outperforming the other two overall. However, SMaSH often demonstrates poor performance for rare cell types. As an example, SMaSH is unable to correctly predict any of the dendritic cells, the second rarest cell type in the PBMC 3k dataset whereas scGIST, scGeneFit, and geneBasis correctly predicted 100%, 89%, and 78% of them respectively.

### Prioritization of easy-to-target genes

scGIST provides the option to prioritize various genes and complexes of interest, such as genes that are easy to target, through user given priority scores. A considerable number of genes are difficult to target in FISH-based technologies due to a number of reasons. First, FISH-based methods use probes to target transcripts. For robust quantification, there needs to be a sufficient number of targetable regions per transcript [28, 29]. Second, targeting specific transcripts becomes challenging for genes with high sequence similarity, e.g., genes in the human olfactory receptor family [30]. Finally, highly expressed genes create optical crowding in the FISH images, making it difficult to target those genes [10]. These challenges, combined with the need for error-correcting barcodes, have so far limited the number of targetable genes to about half of the transcriptome.

To explore this, we experiment with the Mouse Endoderm dataset. Lohoff et al. [31] reported 387 genes that are easier to target and are good at cell type classification for the single-cell molecular map of mouse gastrulation and early organogenesis [32]. Out of these 387 genes, 2 were dropped at the preprocessing step. The remaining 385 genes were fed to our model with priority scores set to 0 and 1 in two experiments.

**Fig. 3:**
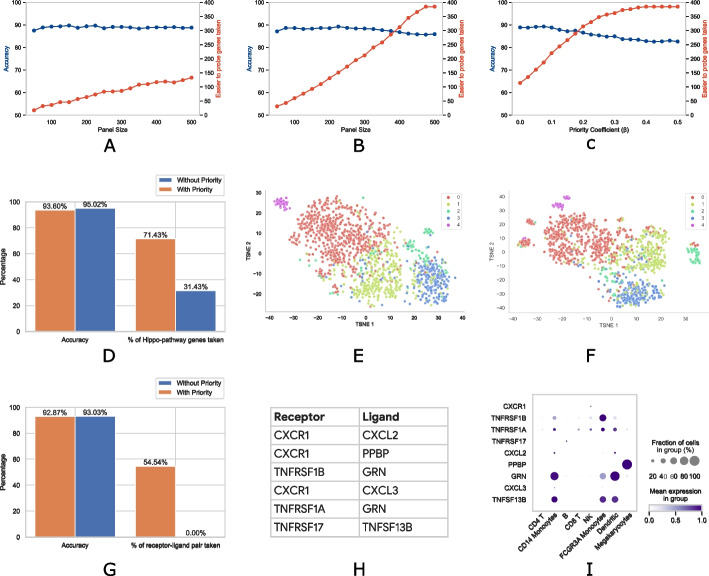
**A**–**C** Analysis of prioritization of easy-to-target genes for the Mouse Endoderm dataset. Accuracy and the numbers of easy-to-target genes included in gene panels when **A** priority scores of all genes = 0, and **B** priority scores of easy-to-target genes = 1, co-efficient of priority ($\beta$) = 0.2 for varying panel sizes, and **C** priority scores of easy to probe genes = 1, panel size = 500 for varying co-efficient of priority ($\beta$). **D**–**F** Analysis of YAP-target genes upon dysregulation of the Hippo signaling pathway on the Head and Neck Cancer dataset. **D** Comparison of accuracy and the number of YAP-target genes taken in a panel of size 60 with and without prioritization. t-SNE plots of Fibroblast subtypes using gene panel of size 60 with **E** YAP-target genes prioritized and **F** YAP-target genes not prioritized. **G**–**I** Receptor-ligand complex analysis on the PBMC 3k dataset. **G** Comparison of accuracy and the number of receptor-ligand pairs taken in a panel of size 60 with and without prioritization. **H** Receptor-ligand complexes included in the panel. **I** Dot plot showing, for each gene encoding receptors or ligands, fractions of cells of each type where they are expressed, and their mean expressions

Figure 3A and B show the accuracy of the method and the numbers of easy-to-target genes included in the panel for priority scores of 0 and 1 respectively for various panel sizes. We observe that when the priority scores are set, many more easy-to-target genes are included in the panel without much reduction in accuracy. The actual numbers are listed in Additional file 1: Tables S9-S11. For example, for panel size 100, when no priority score is set, 89.26% accuracy is achieved while including 36 easy-to-target genes. But when the priority scores are set to 1, 60 such genes are included with accuracy only dropping slightly to 88.64%. Even if we consider a large panel of size of 500, we find that only 133 easy-to-target genes are included in the panel when no priority is set whereas all 385 are selected when priority scores are set to 1.

The importance of the prioritized genes can also be tuned through the co-efficient of priority ($\beta$). Figure 3C and Additional file 1: Table S11 show the accuracy and the numbers of easy-to-target genes included in the panels against varying co-efficient of priority ($\beta$) for panel size of 400. For $\beta =0$, which is equivalent to not prioritizing any gene, we find that 114 of those genes are included in the panel with cell type prediction accuracy of 88.93%. For the default $\beta =0.2$, the number of such genes in the panel increases to 315 with accuracy dropping to 86.57%. Finally, for $\beta \ge 0.4$, all 385 easy-to-target genes are selected in the gene panel.

### Inclusion of other genes of interest

In many experiments, genes of interest, other than the marker genes to identify cell types, need to be included in the panel to address specific biological questions. One approach to this may be to include such genes in the panel first and then select marker genes to fill the remaining slots in the panel. However, this approach may lead to a drastic reduction in cell type prediction accuracy. scGIST provides the option to systematically trade-off between genes-of-interest inclusion and prediction accuracy through the incorporation of user provided priority scores in the loss function.

As a case study, we consider the genes activated by YAP (Yes Associated Protein; an oncogene) upon dysregulation of the Hippo signaling pathway in head and neck cancer-associated fibroblasts (CAFs) [33, 34]. Spatially mapping YAP-target genes in these cells can delineate the different CAF populations and their spatial relationship with other cell types. Thus, we assign 35 YAP-target genes priority scores of 0 and 1 in two experiments. All other genes were given priority scores of 0 in both experiments. We then design panels of size 60 for the Head and Neck Cancer dataset. The accuracy and the percentages of YAP-target genes included in the panel in the two settings are shown in Fig. 3D. We find that when no priority is provided, 31.43% of YAP-target genes are included in the panel and the prediction accuracy is 95.02% for all cell types in the Head and Neck Cancer dataset. However, when they are assigned priorities of 1, 71.43% of those genes are selected and the accuracy only slightly decreases to 93.60%.

Next, we isolate the fibroblast cells in the dataset and identify subtypes within them. We perform principal component analysis (PCA) on the expression values of all genes and construct a graph based on the first 60 principal components. We then run the community detection algorithm Leiden [35] on this graph to cluster the cells which reveal four subtypes (types 0–3). Subsequently, we create a t-SNE plot using the 60 genes selected in the panel when the YAP-target genes are prioritized which is shown in Fig. 3E. We observe that a subset of cells appears to form a separate cluster (labeled Type 4 and shown in a different color). This subset of cells consistently shows up as a different cluster in t-SNE plots with various settings (not shown). However, when a similar t-SNE plot is constructed using the gene panel with YAP-target genes not prioritized, this cluster is split into multiple sub-clusters with some cells grouped with type 0 cells (Fig. 3F). This illustrates that the inclusion of specific genes of interest may provide insights that may not be obtained from marker genes in the original dataset because expressions of marker genes may not correlate well with that of the genes of interest in question.

If addressing a biological question necessitates the inclusion of all the genes in the pathway, this can be done by gradually increasing the co-efficient of priority $\beta$ until all such genes are included in the panel. To illustrate this, we kept the priority scores of YAP-target genes at 1 but gradually increased $\beta$ from 0.05 to 0.5, which progressively increased the number of those genes taken. All 35 YAP-target genes were taken when the value of $\beta$ was greater than or equal to 0.45 with only a slight reduction in accuracy (Additional file 1: Fig. S6).

### Inclusion of complexes of interest (receptor-ligand complexes)

scGIST also permits researchers to prioritize genes encoding various protein complexes such as receptor-ligand complexes. Cell-cell interactions drive cell differentiation and organ development, and disruption in signaling pathways has been implicated in various diseases [36]. Receptor-ligand complexes are key to understanding intercellular communication, and as a result, it is important to measure their expression, especially at the surfaces of tissues. However, these genes are often not included in gene panels as they are not markers for cell types or are expressed in a small number of cells.

To analyze this, we collected 11 receptor-ligand complexes from CellPhoneDB [37]. We then encoded them using pairs (see the “Methods” section for details) and experimented with the PBMC 3k dataset. The results are summarized in Fig. 3G. We observe that the panel includes none of the receptor-ligand pairs when the complexes are not prioritized. However, when they are prioritized, as many as 54.54% of the pairs are selected in the panel. Moreover, this is at the expense of only a small decrease in accuracy from 93.03 to 92.87%.

The receptor-ligand complexes that are included in the gene panel are provided in Fig. 3H while a dot plot of expressions of the genes by cell types is shown in Fig. 3I. The dot plot illustrates, for each cell type, the fraction of cells where each of those genes are expressed and their mean expressions. We observe that the gene *PPBP* appears to be only expressed in Megakaryocytes and hence is a good candidate as a marker. However, some genes such as *TNFRSF1A* and *TNFRSF1B* are expressed in a number of cell types while *CXCR1* is expressed highly in only a small fraction of NK cells. Similarly, *CXCL2* is expressed highly in a small number of CD14 Monocytes and Dendritic cells. scGIST is able to include these genes in the panel based on user prioritization and thus potentially providing insights that would be missed otherwise.

We then explored the performance of scGIST on the inclusion of multi-gene complexes. However, our search revealed only one such complex of three genes in CellPhoneDB relevant to the four datasets we analyzed. We created ${{3}\atopwithdelims (){2}} =3$ pairs and designed gene panels for the Tabula Sapiens dataset with and without prioritizing the complex. The results are shown in Additional file 1: Fig. S7. When the complex is not prioritized, it is not included in the panel resulting in an accuracy of 92.18%. But when the complex is assigned a priority of 1, it is included in the panel with accuracy reducing to 92.16%. Next, we created 6 additional simulated complexes by randomly grouping 3–5 genes. When the complexes are prioritized, all 7 complexes are included in the panel in contrast to none when they are not prioritized with accuracy dropping from 92.14 to 91.06%. While scGIST does not guarantee that all genes in a prioritized complex will be included in the panel, a trade-off between accuracy and the inclusion of genes in the complexes can be achieved by varying the co-efficient of priority ($\beta$) (Additional file 1: Fig. S8).

### Evaluation on a spatial transcriptomics dataset

Finally, we benchmark scGIST on a spatial transcriptomic dataset. We analyzed a Mouse Olfactory seqFISH+ dataset [17] for which an scRNA-seq dataset [38] is also available. The seqFISH+ dataset contains 10,000 genes whereas the scRNA-seq dataset contains 27,998 genes. The datasets were first modified to retain only the 9907 genes present in both the datasets. We then created gene panels using scGIST from the scRNA-seq dataset and predicted the cell types on the seqFISH+ dataset based on those gene panels. A similar analysis was performed using geneBasis.

**Fig. 4:**
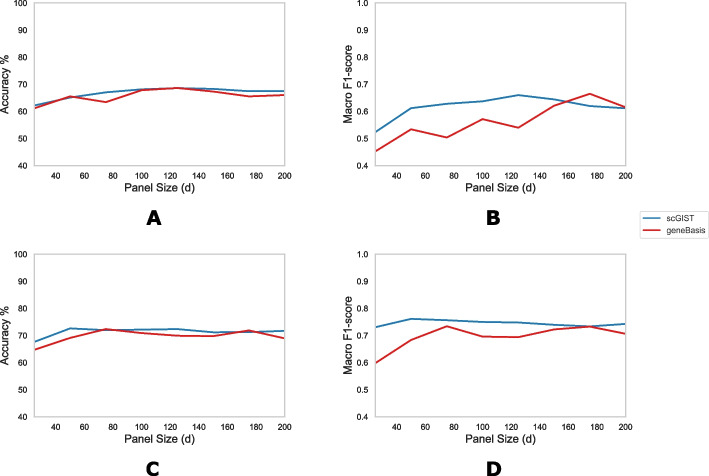
Evaluation of methods on a Mouse Olfactory seqFISH+ dataset. **A** Accuracy and **B** macro F1-scores against panel sizes for scGIST and geneBasis when gene panels are designed using an scRNA-seq dataset and cell types are predicted on the seqFISH+ dataset based on the gene panels. **C** Accuracy and **D** macro F1-scores against panel sizes for scGIST and geneBasis when gene panels are designed using the seqFISH+ dataset and cell types are predicted on seqFISH+ test data

Figure 4A and B show the accuracies and macro F1-scores, respectively, for panel sizes varying from 25 to 200 at increments of 25 for both scGIST and geneBasis. The values for scGIST are averages over 5 runs for each panel size. The accuracies were measured using k-nearest neighbor (k-NN) similarly to other analyses as described in the “Methods” section. We observe that scGIST generally outperforms geneBasis both in terms of accuracy and macro F1-score. However, we find that for both scGIST and geneBasis, the accuracies and macro F1-scores are lower than those while predicting cell types using scRNA-seq datasets.

To explore this issue, we next created gene panels from the seqFISH+ dataset itself and predicted the cell types on the seqFISH+ test data using the gene panels designed by scGIST and geneBasis. The accuracies and macro F1-scores for varying panel sizes are shown in Fig. 4C and D, respectively. We observe that again the accuracies and macro F1-scores are lower compared to those for scRNA-seq datasets. This indicates that there is more variability in seqFISH+ expression estimates than scRNA-seq ones which constraints the accuracy with which cell types can be predicted using seqFISH+ datasets.

## Discussion

By adopting a powerful neural network architecture for feature selection and a custom loss function, we have shown that scGIST is an effective and broadly applicable tool for sc-ST panel design. The model optimizes cell type detection accuracy while satisfying users’ need regarding a gene set of interest and staying within the size limit of the sc-ST gene panel.

We have demonstrated scGIST’s efficacy on four datasets and under different use cases. We believe that scGIST owes its success as much to its architecture and the loss function as to the inherent covariance structures of gene expression relationships in single-cell data. Recent works from Aviv Regev and colleagues [6, 39] have shown that genes in these datasets form co-expression modules—the expression of any gene from a module is generally sufficient to recover the expression of other genes in that module. We believe that scGIST can successfully leverage this property to select genes satisfying various constraints without sacrificing classification accuracy.

Importantly, scGIST has consistently outperformed the alternative tools [20–22] that can be used to derive marker gene panels under size constraints. While it is difficult to give theoretical arguments that place one method clearly as the best method, one of the strengths of scGIST over other methods is it explicitly incorporates the panel size as a constraint in the regularization function. Moreover, whereas scGeneFit relies on linear programming, scGIST utilizes a non-linear neural network allowing it to explore a larger function space. Although SMaSH [22] is a deep neural network like scGIST, SMaSH requires post hoc model interpretation methods to rank the features and applies a greedy feature selection strategy that does not take the panel size into account. In contrast, scGIST integrates feature ranking and all other constraints including the panel size into the loss function. We believe this helps scGIST make the necessary feature trade-offs directly during model optimization and select the most appropriate features that maintain accuracy while satisfying the other constraints as much as possible. In comparison to geneBasis [21], scGIST does not require greedy feature selection, i.e., taking the gene that leads to the most improvement in results at each step as long as the panel is not full, and need not make assumptions about cell-to-cell distance metrics—both characteristics should have helped scGIST to outperform geneBasis.

One potential limitation of scGIST is, similarly to the aforementioned methods, it builds gene panels for spatial transcriptomics using scRNA-seq data. The panels designed by these tools will be of limited use if the two technologies differ substantially in their transcript detection efficiency. Our experiments indicate that scGIST outperforms other tools while predicting cell types on spatial transcriptomics datasets using gene panels designed based on scRNA-seq datasets. Moreover, recent literature suggests that the two technologies generally agree in transcript detection and quantification. Correlations between MERFISH/Vizgen/seqFISH+ data and RNA-seq FPKM values were shown to be as high as 0.94 [40] and are generally around 0.8 [17, 28, 41, 42]. Although two studies reported correlations of 0.5 and 0.6 [29, 43], this is largely due to a drop in correlations for genes expressed at low levels [42]. To test if scGIST is biased towards genes with low transcript counts, we analyze its performance excluding the genes with expressions in the lowest ten percentile. The results (Additional file 1: Fig. S9) show that the accuracy of scGIST does not decrease substantially even if we exclude the genes with very low expression levels. To ensure scGIST’s applicability, it may be run by excluding or de-prioritizing genes with very low transcript counts. Overall, we find that the gene panels designed based on scRNA-seq datasets are informative and can successfully distinguish different cell types in spatial transcriptomics experiments. However, gene panels designed from scRNA-seq datasets cannot be perfect for FISH-based assays due to technological differences, and therefore, these gene panels may need to be verified manually based on domain knowledge.

scGIST has opened up several exciting directions for future research. First, currently all methods, including scGIST, design panels for a single biological context, e.g., a particular tissue type or a particular timepoint. An impactful direction would be to design panels that maximize the use of the selected genes (i.e., their probes) for multiple biological contexts. Secondly, now that scGIST enables biologists to specify gene priorities, we anticipate there will be more research on modeling the ease of probe design for given transcript sequences [3]. Finally, our future work will focus on implementing scGIST as an interactive tool for panel design, where the model will explain why it could not satisfy all constraints and what potential trade-offs might be necessary to optimize a particular goal, e.g., including all ligand-receptor complexes.

## Methods

### scGIST model

scGIST takes as input *N* instances $\{(\textbf{x}_{\textbf{1}}, y_1), (\textbf{x}_{\textbf{2}}, y_2), \dots , (\textbf{x}_{\textbf{N}}, y_N) \}$ where $\textbf{x}_{\textbf{i}}\in \mathbb {R}^n$ is log normalized gene expression values of *n* genes of the *i*-th cell and $y_i\in \{1,2,\dots , c\}$ is the corresponding cell label. It learns a deep neural network model from the training instances to predict cell labels *y* from gene expression values $\textbf{x}$ and uses a special regularization function to ensure that only a subset of the genes is used for prediction [19].

The deep neural network model is shown in Fig. 1B. Let $\textbf{x}=(x_1,x_2,\dots , x_n)$ be the inputs to the network. To perform feature selection, we add a one-to-one linear layer following the input layer. If the weights of the one-to-one linear layer are $\textbf{w} = (w_1, w_2, \dots , w_n)$, then the output of the layer will be $\mathbf {w.x} = (w_1 x_1, w_2 x_2, \dots , w_n x_n)$. Here $\textbf{w}$ has to be a sparse binary vector in order to perform feature selection.

Following the one-to-one layer, there are 2 fully connected layers with 32 and 16 neurons respectively with ReLU activation and L2 regularization functions. Finally, there is an output layer with a soft-max activation function having *c* neurons. We denote the parameters of these layers by $\textbf{W}^{\mathbf {(k)}}$ and $\textbf{b}^{\mathbf {(k)}}$ for $k=1,2,3$, where $\textbf{W}^{\mathbf {(k)}}$ is the weight matrix of the layer and $\textbf{b}^{\mathbf {(k)}}$ is the corresponding bias vector. So, the parameters of the model can be denoted together as $\theta = \{\textbf{w}, \textbf{W}^{\mathbf {(1)}}, \textbf{b}^{\mathbf {(1)}}, \textbf{W}^{\mathbf {(2)}}, \textbf{b}^{\mathbf {(2)}}, \textbf{W}^{\mathbf {(3)}}, \textbf{b}^{\mathbf {(3)}}\}$.

To learn the model parameters, we have to minimize the following loss function:

$$\begin{aligned} \min _\theta J(\theta ) = l(\theta ) + \lambda _0 R + \lambda _1 \sum _{k = 1}^{3} ||\textbf{W}^{\mathbf {(k)}}||_2 \end{aligned}$$

Here, $l(\theta )$ is the log-likelihood of the given data which can be written as

$$\begin{aligned} l(\theta ) = - \frac{1}{N} \sum _i^N \log p(y_i | h^{(2)}(\textbf{x}_{\textbf{i}})) \end{aligned}$$

where $\varvec{h^{(2)}(\textbf{x}_{\textbf{i}})}$ is the output of the second fully connected layer given input $\textbf{x}_{\textbf{i}}$. The weights are learnt by minimizing the loss function and the genes corresponding to the *d* highest weights in the feature selection layer are selected as the panel.

$\varvec{R}$ is a special regularization term in the one-to-one layer that is used for feature selection which is described in the next section. $\lambda _1\sum _{k = 1}^{3} ||W^{(k)}||_2$ is used for L2 regularization of the final three layers. This is used to reduce bias and to make sure that no small value of $\varvec{w}$ in the one-to-one layer is amplified by any of $\textbf{W}^{\mathbf {(k)}}$ in the subsequent layers.

### Regularization

In order to make $\textbf{w}$ a sparse binary vector to perform feature selection, and to prioritize the inclusion of user specified genes and complexes, we include the following special regularization in the loss function:

$$\begin{aligned} R = \sum _{i=1}^{n} |w_i| |w_i - 1| + \alpha |||\textbf{w}||_1 - d| + \beta \sum _{i\in \mathcal {G}} p_i (1 - \min (|w_i|, 1)) + \gamma \sum _{(i, j) \in \mathcal {C}} ||w_i| - |w_j|| \end{aligned}$$

where

1. The first term $(\sum _{i=1}^{n} |w_i| |w_i - 1|)$ drives the weights $w_i$ towards either 0 or 1. We can see from Fig. 1C that the error is minimum (0) when either $w_i = 0$ or $w_i = 1$ for $i=1, \dots , n$. Thus, this regularization term promotes the sparsity of the weights.
2. $\alpha |||w||_1 - d|$ makes sure that the number of genes used to predict cell labels is equal to *d*, the target gene panel size. As each $|w_i|$ is close to 0 or 1, the sum indicates the number of features taken. We will assign a penalty if the number of features taken is more or less than *d*. Here $\alpha$ is a hyperparameter that determines how much the model is penalized if it takes more or less than *d* parameters. We have found empirically that setting $\alpha =1.5$ provides satisfactory performance. Alternatively, this term can be set in a way to penalize the model only if the number of features exceeds *d* (Additional file 1: Supplementary Note 3.1).
3. $\beta \sum _{i\in \mathcal {G}} p_i (1 - \min (|w_i|, 1))$ is an additional term that makes the model prioritize the inclusion of genes of interest. Here $\mathcal {G}$ is the set of user provided genes of interest and $p_i$ is the priority score of the *i*-the gene. If $p_i > 0$, we apply an additional penalty if that gene is not included in the panel (Fig. 1E). The amount of penalty is proportional to the priority of the gene. Here $\beta$ indicates how much significance we put on selecting the genes of interest. There is a trade-off between accuracy and how many genes of interest are taken because these genes may not provide much information for classification.
4. Finally, the term $\gamma \sum _{(i, j) \in \mathcal {C}} ||w_i| - |w_j||$ is used for inclusion of complexes. Here $\mathcal {C} = \{(i_1, j_1), (i_2, j_2), \dots \}$ is a set of pairs of genes that represents complexes of interest. These complexes can be provided by users to indicate that either both genes from a pair or none should be selected. The regularization function will assign a penalty if two genes of a pair do not have the same weights. By default, one of the two genes in the pair is randomly assigned a priority score of 1. However, the user has the option of assigning priority scores to both of the genes. If there are complexes with more than two interacting genes, they can be encoded by enumerating all pairs in the complex.

### Datasets

To evaluate our method, we have used the following datasets:

*PBMC3k*: This is a dataset of peripheral blood mononuclear cells (PBMCs) [44] from a healthy donor freely available from 10x genomics (https://support.10xgenomics.com/single-cell-gene-expression/datasets/1.1.0/pbmc3k). PBMCs are primary cells with relatively small amounts of RNA ($\sim 1$pg RNA/cell). There are 2700 single cells that were sequenced on the Illumina NextSeq 500. The number of genes is around 1800 after preprocessing. There are 8 cell types present in the dataset.

*Head and Neck Cancer*: The dataset contains transcriptomes of 6000 single cells from 18 head and neck squamous cell carcinoma (HNSCC) patients, including five matched pairs of primary tumors and lymph node metastases which is collected by Puram et al. [24]. There are 9 cell types present with imbalanced distribution.

*Tabula Sapiens (Lungs)*: The Tabula Sapiens Dataset [25] is a human reference atlas that contains around 500,000 cells from 24 different tissues and organs, many from the same donor. This atlas enabled molecular characterization of more than 400 cell types, their distribution across tissues, and tissue-specific variation in gene expression. We have used a subset of this dataset consisting of cells only from lungs which contains 14 cell types.

*Mouse Endoderm Dataset*: This is a dataset of single-cell transcriptomes representing all endoderm populations within the mouse embryo until midgestation to delineate the ontogeny of the mammalian endoderm [26]. The dataset contains more than 110,000 single-cell transcriptomes. We have used a subset of this dataset containing 20,000 cells and 23 cell types.

*Mouse Olfactory Datasets*: The scRNA-seq mouse olfactory dataset [38] contains 20,354 cells and 27,998 genes for 7 different cell types. The seqFISH+ mouse olfactory dataset [17] contains 2050 cells and 10,000 genes for 9 cell types. We have used the 9907 genes that are common in both datasets for our experiment.

### Data preprocessing

All the datasets were preprocessed using SCANPY [45] by following a standard pre-processing workflow for scRNA-seq data in Seurat [46]. First, a basic filtering was applied where any cells with less than 200 genes or any genes expressed in less than 3 cells were discarded. Total-count normalization and logarithmization were then performed on the datasets. Highly variable genes were subsequently identified and the rest of the genes were discarded. Effects of total counts per cell and the percentage of mitochondrial genes expressed were regressed out. Each gene expression was finally scaled to unit variance and values exceeding standard deviation 10 were clipped at 10. In addition, a maximum of 1000 cells per cell type were retained.

### Evaluation

To evaluate the performance of different marker gene panel selection methods, we followed the approach used to evaluate SMaSH [22]. Each of them was executed to find a marker gene panel of size *d*. Only the selected genes were kept in the dataset, i.e., all other genes that are not present in the marker gene panel were removed from the dataset. So the new dataset was of dimension $n \times d$ where *n* is the number of cells in the dataset. The new dataset was split into train and test sets in 75:25 ratio. Then a k-nearest neighbor (k-NN) classifier was trained using the training dataset. After that accuracy and macro F1 score (Additional file 1: Supplementary Note 3.2) was measured on the test dataset. This accuracy and macro F1-score are used in this paper to compare different methods.

### Supplementary information

**Additional file 1.** Supplementary information for scGIST: gene panel design for spatial transcriptomics with prioritized gene sets. This fle includes 9 supplementary figures, 11 supplementary tables and 3 supplementary notes containing 3 figures relevant to the article and referenced in the publication.

**Additional file 2.** Peer review history.

## Data Availability

scGIST is implemented in Python and its source code is publicly available under the MIT license at Zenodo [47] and GitHub [48](https://github.com/yafi38/scGIST). PBMC 3k [44] is available for download through the 10x Genomics website. Head and Neck Cancer [24] is available through the Gene Expression Omnibus, under accession number GSE103322. Tabula Sapiens [25] is available for download through the Tabula Sapiens website. Mouse Endoderm [26] is available through the Gene Expression Omnibus, under accession number GSE123046. The processed mouse olfactory scRNA-seq dataset [38] and seqFISH+ dataset [17] were obtained from the codebase (https://github.com/MarioniLab/am_geneBasis) of geneBasis [21].
