# Supplementary material for: scGIST: gene panel design for spatial transcriptomics with prioritized gene sets
Source: Genome Biol. 2024 Feb 26;25:57. doi: 10.1186/s13059-024-03185-y (PMC10895727; doi:10.1186/s13059-024-03185-y)
Supplement: Supplementary file 1 — Additional file 1. Supplementary information for scGIST: gene panel design for spatial transcriptomics with prioritized gene sets. This fle includes 9 supplementary figures, 11 supplementary tables and 3 supplementary notes containing 3 figures relevant to the article and referenced in the publication. [file 13059_2024_3185_MOESM1_ESM.pdf]

Supplementary information for scGIST: gene panel design for spatial transcriptomics with prioritized gene sets

Mashrur Ahmed Yafi[1], Md. Hasibul Husain Hisham[1], Francisco Grisanti[2], James F. Martin[2,3], Atif Rahman[1,*], and Md. Abul Hassan Samee[2,*]

[1]Department of Computer Science and Engineering, Bangladesh University of Engineering and Technology, Dhaka, 1205, Bangladesh
[2]Department of Integrative Physiology, Baylor College of Medicine, Houston, TX 77030, USA
[3]Cardiomyocyte Renewal Laboratory, Texas Heart Institute, Houston, TX 77030, USA
[*]Co-corresponding authors: atif@cse.buet.ac.bd, samee@bcm.edu

Contents

1 Supplementary Figures **3**

2 Supplementary Tables **11**

3 Supplementary Notes **18**

3.1 Alternate regularization penalty for panel size 18

3.2 Evaluation metrics 19

3.3 Accuracies and Macro F1-Scores from Multiple Runs 19

1 Supplementary Figures

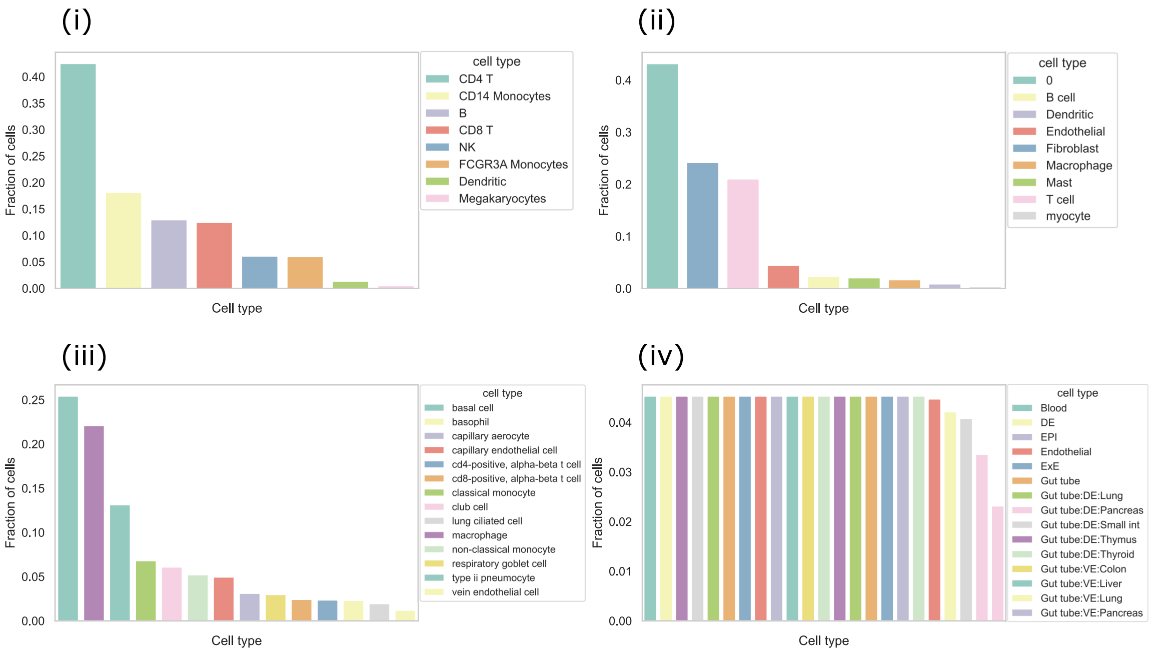

Figure S1: Ordered bar plot representing relative cell type abundance in different dataset. Panel **(i)** PBMC 3K, **(ii)** Head and Neck Cancer, **(iii)** Tabula Sapiens, **(iv)** Mouse Endoderm.

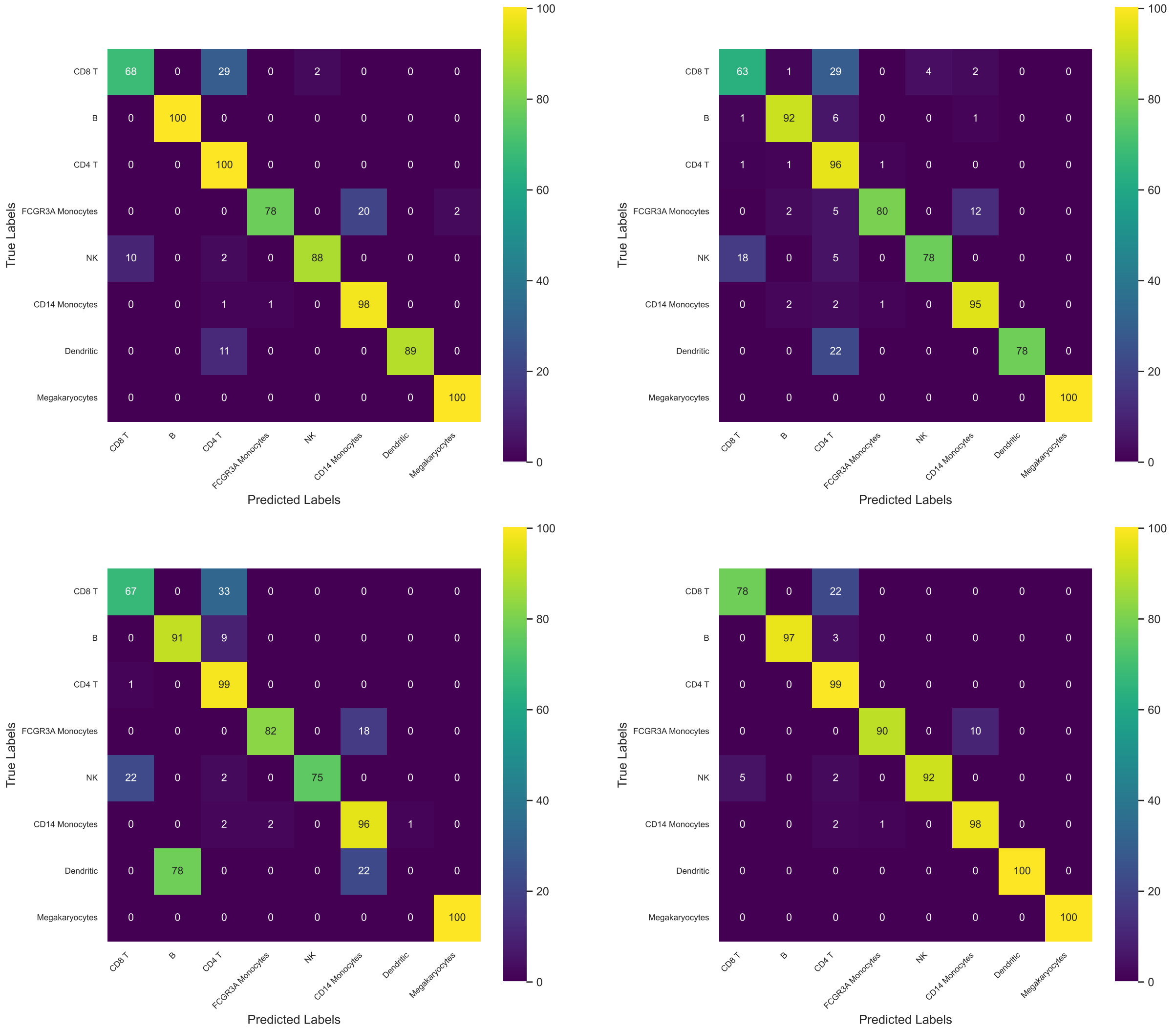

Figure S2: Confusion matrices of (a) scGeneFit, (b) geneBasis, (c) SMaSH and (d) scGIST on the PBMC 3K Dataset for panel size of 60.

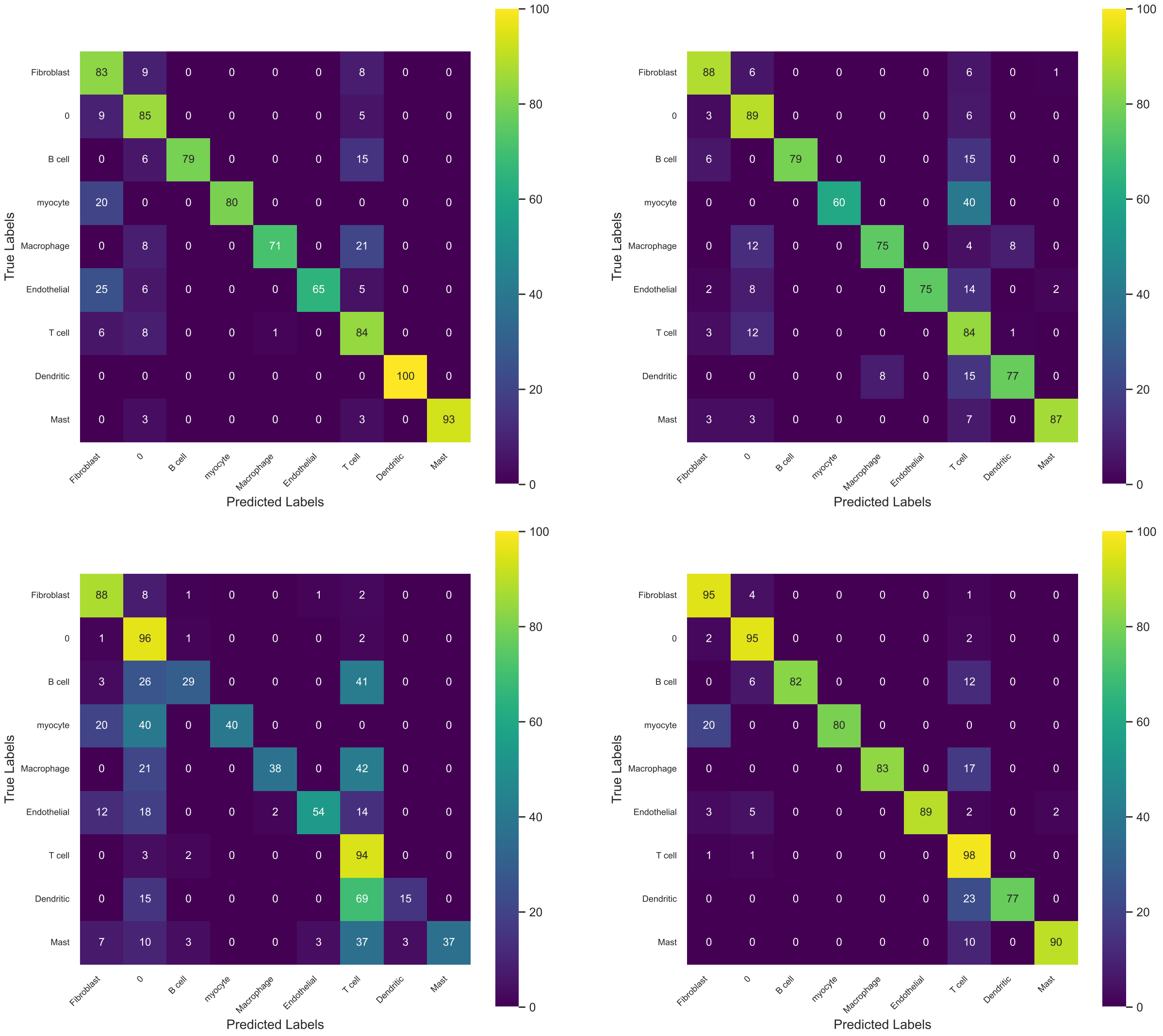

Figure S3: Confusion matrices of (a) scGeneFit, (b) geneBasis, (c) SMaSH and (d) scGIST on the Head and Neck Cancer Dataset for panel size of 60.

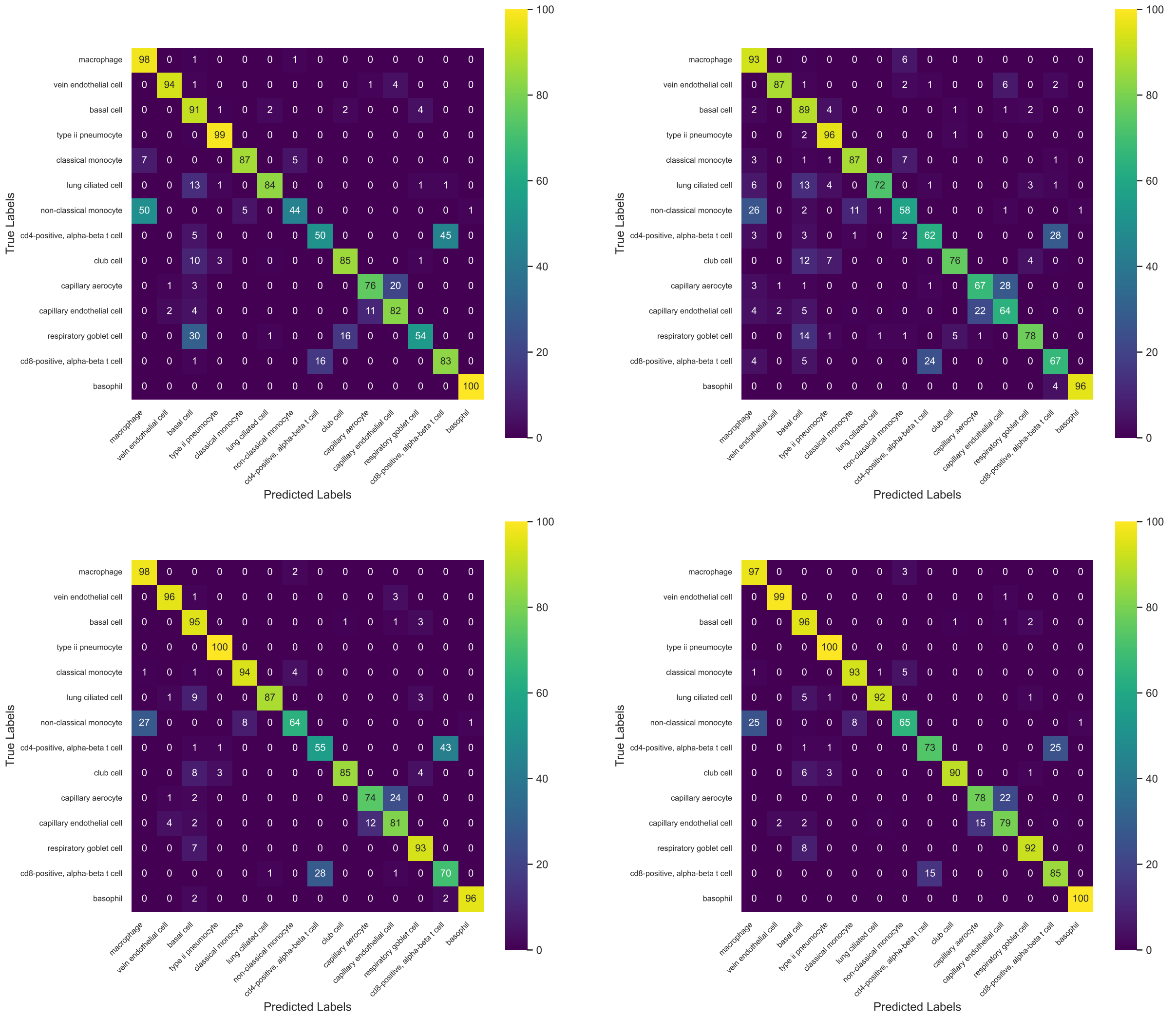

Figure S4: Confusion matrices of (a) scGeneFit, (b) geneBasis, (c) SMaSH and (d) scGIST on the Tabula Sapiens Dataset for panel size of 60.

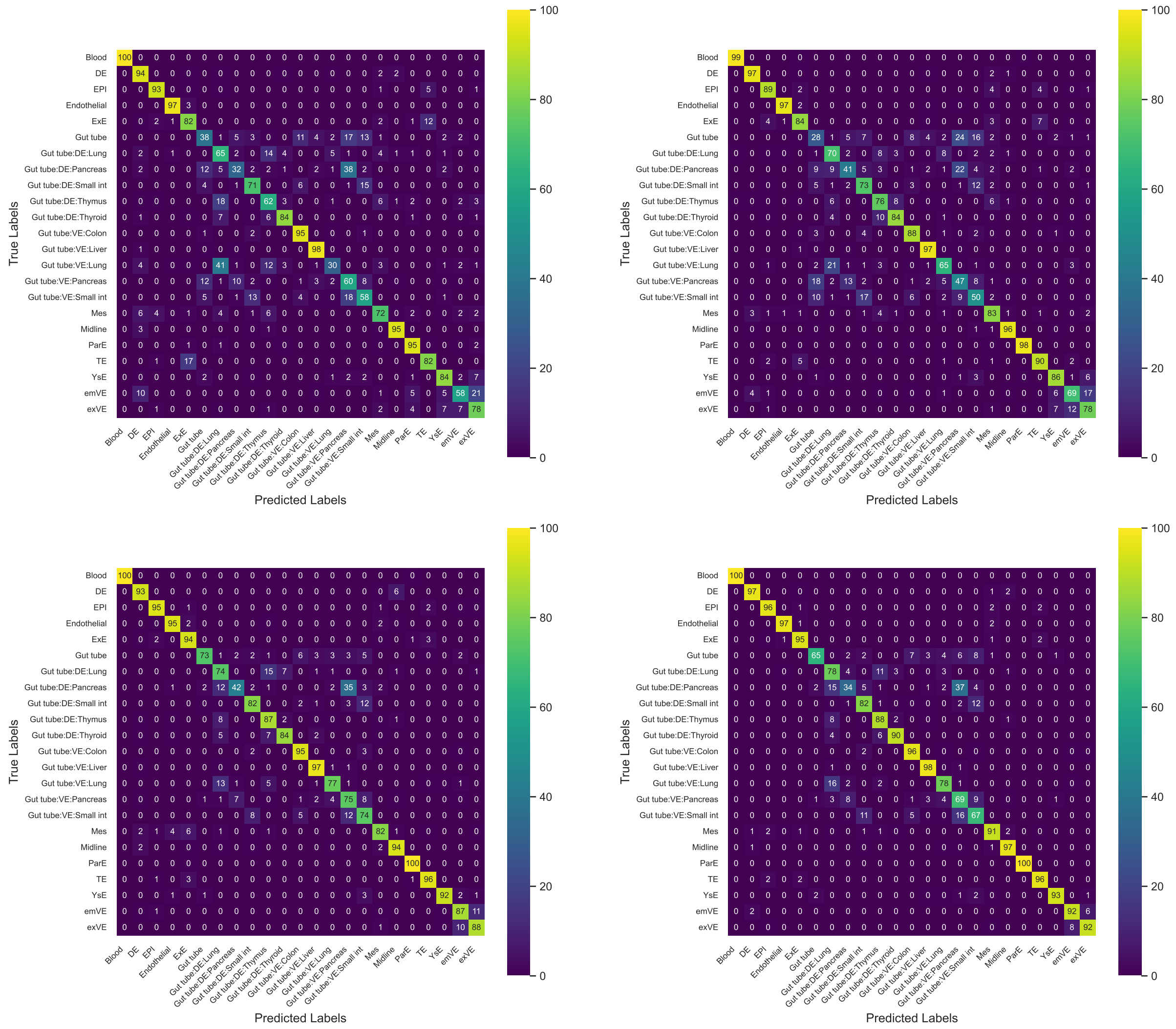

Figure S5: Confusion matrices of (a) scGeneFit, (b) geneBasis, (c) SMaSH and (d) scGIST on the Mouse Endoderm Dataset for panel size of 60.

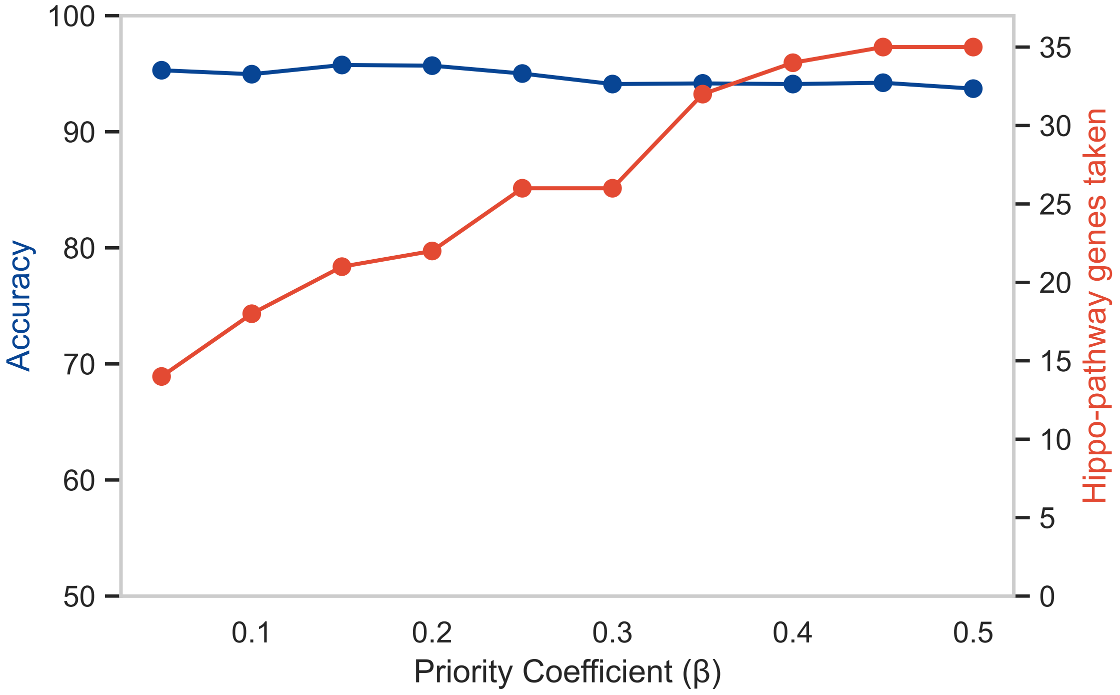

Figure S6: Comparison of accuracy and the number of YAP-target~~Hippo pathway~~ genes taken vs co-efficient of priority (β) in a panel of size 100.

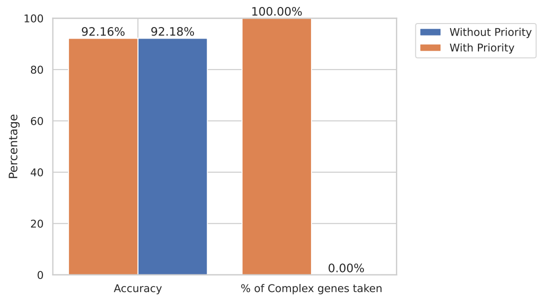

Complex Name	Gene Name
IL2_receptor_HA	IL2RA
	IL2RB
	IL2RG

Figure S7: Complexes included in the panel from Tabula Sapiens Dataset

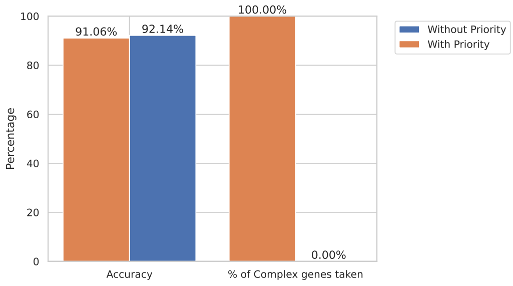

(a)

Complex Name	Gene Name
IL2_receptor_HA	IL2RA
	IL2RB
	IL2RG
Simulated_complex_A	AC104248.1
	RPL35A
	LINC01871
Simulated_complex_B	BPIFB1
	LINC00891
	HPGD
Simulated_complex_C	ZNF655
	BNIP2
	LINC01550
	PDIA5
Simulated_complex_D	ILF2
	HLA-DQA1
	PPM1K
	TYROBP
Simulated_complex_E	COL9A2
	Z99774.1
	ZNF570
	PELI2
	AC104024.1
Simulated_complex_F	ARL8A
	NR2C2
	ARSB
	NPM1P18
	CTSA

(b) Complexes

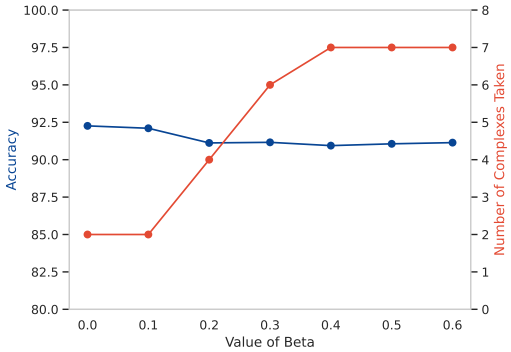

(c)

Figure S8: **(a-c).** Analysis of more complex inclusion in a panel size of 80. **a.** Comparison of accuracy and number of complexes taken in the panel. **b.** All the complexes considered for the experiment, including 1 real receptor-ligand complex and 6 simulated ones. **c.** Accuracy and number of complexes inclusion for varying co-efficient of priority (β).

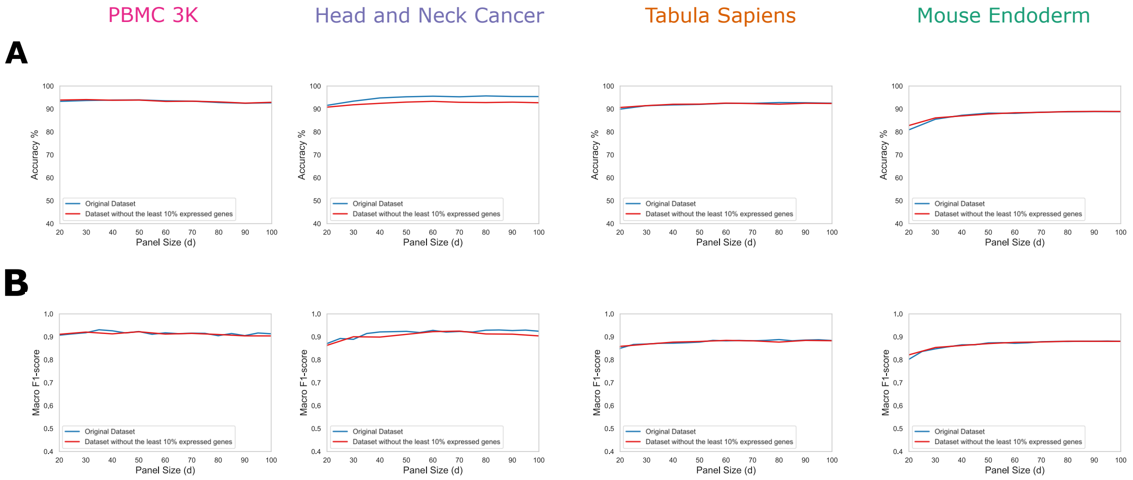

Figure S9: Comparison of **A.** Accuracies and **B.** Macro F1-Scores between the gene panels created using scGIST from the original datasets and datasets without the lowest 10% expressed genes.

2 Supplementary Tables

Panel size	scGeneFit	geneBasis	SMaSH	scGIST
15	85.30	85.00	91.21	92.10
20	88.94	85.00	92.36	93.31
25	90.30	86.36	92.88	93.43
30	92.42	86.36	93.58	93.69
35	92.73	85.76	93.55	93.89
40	93.64	85.61	93.45	93.89
45	93.94	86.97	93.03	93.56
50	93.48	87.42	93.15	93.94
55	93.79	89.24	92.82	93.36
60	93.33	89.09	92.45	93.56
65	92.73	88.79	92.06	93.38
70	92.73	87.27	92.24	93.41
75	93.33	86.21	92.18	92.85
80	93.64	86.82	92.39	92.80
85	92.88	85.91	92.30	93.06
90	93.03	85.76	91.97	92.50
95	92.88	86.06	91.91	92.98
100	91.97	85.76	91.70	92.68

Table S1: Accuracy of various methods on the PBMC Dataset

Panel size	scGeneFit	geneBasis	SMaSH	scGIST
15	0.81	0.79	0.85	0.91
20	0.84	0.79	0.86	0.91
25	0.85	0.82	0.87	0.91
30	0.91	0.85	0.90	0.92
35	0.91	0.84	0.90	0.93
40	0.91	0.83	0.90	0.93
45	0.92	0.86	0.90	0.92
50	0.91	0.86	0.90	0.92
55	0.92	0.88	0.90	0.91
60	0.91	0.88	0.88	0.92
65	0.89	0.86	0.88	0.91
70	0.92	0.84	0.89	0.92
75	0.91	0.82	0.90	0.92
80	0.93	0.83	0.90	0.90
85	0.92	0.82	0.90	0.91
90	0.93	0.82	0.89	0.91
95	0.93	0.83	0.89	0.92
100	0.92	0.82	0.89	0.91

Table S2: Macro F1-score of various methods on the PBMC Dataset

Panel size	scGeneFit	geneBasis	SMaSH	scGIST
15	44.22	63.48	79.38	89.54
20	53.00	67.04	80.89	91.57
25	54.64	69.42	82.14	92.67
30	55.66	74.35	82.89	93.41
35	54.87	76.84	83.58	94.41
40	61.83	72.54	83.59	94.80
45	61.78	80.01	84.07	94.76
50	77.18	82.67	84.25	95.31
55	77.07	84.77	84.12	95.20
60	83.52	86.07	84.14	95.56
65	84.03	86.47	84.19	95.40
70	85.90	86.41	84.16	95.31
75	86.52	87.54	84.37	95.37
80	87.43	88.56	84.94	95.69
85	88.45	88.62	85.41	95.56
90	91.17	88.67	85.19	95.44
95	91.85	89.30	85.16	95.48
100	92.47	89.86	85.06	95.41

Table S3: Accuracy of various methods on the Head and Neck Cancer Dataset

Panel size	scGeneFit	geneBasis	SMaSH	scGIST
15	0.69	0.53	0.40	0.81
20	0.70	0.61	0.44	0.87
25	0.74	0.64	0.46	0.89
30	0.75	0.75	0.50	0.89
35	0.74	0.76	0.51	0.91
40	0.74	0.75	0.52	0.92
45	0.74	0.78	0.53	0.92
50	0.80	0.79	0.54	0.92
55	0.79	0.81	0.53	0.92
60	0.83	0.81	0.55	0.93
65	0.83	0.83	0.54	0.92
70	0.75	0.84	0.56	0.92
75	0.74	0.85	0.57	0.92
80	0.78	0.86	0.58	0.93
85	0.78	0.86	0.58	0.93
90	0.80	0.87	0.57	0.93
95	0.80	0.88	0.57	0.93
100	0.80	0.88	0.58	0.92

Table S4: Macro F1-score of various methods on the Head and Neck Cancer Dataset

Panel size	scGeneFit	geneBasis	SMaSH	scGIST
15	43.44	72.42	84.18	86.88
20	51.78	77.01	86.98	89.93
25	62.87	78.01	87.86	91.17
30	71.56	79.04	88.38	91.43
35	84.91	82.09	88.70	91.66
40	87.07	82.09	89.42	91.76
45	86.67	82.55	90.24	91.80
50	87.29	83.44	90.93	92.00
55	88.25	84.12	91.25	92.58
60	88.37	85.37	91.47	92.47
65	88.49	85.72	91.69	92.62
70	87.90	86.80	91.94	92.44
75	88.42	86.99	91.98	92.58
80	88.56	87.24	92.05	92.80
85	88.56	86.53	92.00	92.39
90	88.00	86.48	91.99	92.71
95	88.79	86.80	91.60	92.69
100	88.05	86.40	91.77	92.50

Table S5: Accuracy of various methods on the Tabula Sapiens Dataset

Panel size	scGeneFit	geneBasis	SMaSH	scGIST
15	0.46	0.64	0.70	0.80
20	0.57	0.71	0.76	0.85
25	0.67	0.73	0.77	0.87
30	0.73	0.73	0.78	0.87
35	0.80	0.74	0.79	0.87
40	0.80	0.75	0.81	0.87
45	0.80	0.75	0.82	0.87
50	0.80	0.77	0.84	0.88
55	0.81	0.78	0.85	0.88
60	0.81	0.79	0.85	0.88
65	0.82	0.79	0.86	0.88
70	0.81	0.80	0.87	0.88
75	0.82	0.81	0.87	0.88
80	0.82	0.82	0.87	0.89
85	0.82	0.80	0.87	0.88
90	0.82	0.81	0.87	0.89
95	0.83	0.81	0.86	0.89
100	0.82	0.80	0.87	0.88

Table S6: Macro F1-score of various methods on the Tabula Sapiens Dataset

Panel size	scGeneFit	geneBasis	SMaSH	scGIST
15	42.62	56.02	67.25	77.43
20	43.78	61.61	73.99	80.91
25	45.39	66.76	78.91	84.31
30	52.54	69.93	81.68	85.52
35	58.39	71.91	83.94	86.38
40	66.35	72.72	85.26	87.25
45	68.28	74.57	86.29	87.35
50	73.57	76.73	86.76	88.19
55	75.08	77.34	87.10	88.17
60	75.31	77.99	87.56	88.08
65	76.95	79.10	87.61	88.19
70	78.42	79.53	88.05	88.60
75	79.82	79.58	88.32	88.75
80	80.98	80.29	88.36	88.76
85	80.50	80.55	88.58	88.90
90	81.05	81.26	88.70	88.87
95	81.44	81.32	88.75	89.03
100	81.63	81.17	89.07	88.83

Table S7: Accuracy of various methods on the Mouse Endoderm Dataset

Panel size	scGeneFit	geneBasis	SMaSH	scGIST
15	0.41	0.55	0.66	0.77
20	0.42	0.60	0.73	0.80
25	0.44	0.66	0.78	0.84
30	0.51	0.69	0.81	0.85
35	0.57	0.71	0.83	0.86
40	0.65	0.72	0.85	0.87
45	0.67	0.74	0.86	0.87
50	0.72	0.76	0.86	0.87
55	0.74	0.76	0.86	0.87
60	0.74	0.77	0.87	0.87
65	0.76	0.78	0.87	0.87
70	0.77	0.79	0.87	0.88
75	0.79	0.78	0.88	0.88
80	0.80	0.79	0.88	0.88
85	0.79	0.80	0.88	0.88
90	0.80	0.80	0.88	0.88
95	0.80	0.80	0.88	0.88
100	0.80	0.80	0.88	0.88

Table S8: Macro F1-score of various methods on the Mouse Endoderm Dataset

Panel size	Accuracy	No. genes with easy to design probes taken
50	87.52	17
75	88.87	32
100	89.26	36
125	89.40	46
150	89.76	46
175	88.75	57
200	89.41	64
225	89.70	73
250	88.55	83
275	89.11	84
300	89.14	86
325	88.91	95
350	88.39	108
375	88.78	110
400	88.94	117
425	88.87	119
450	89.05	116
475	88.67	124
500	88.81	133

Table S9: Accuracy and numbers of genes with easy to design probes included in the panel by scGIST when no genes are assigned priority and $\beta = 0.2$

Panel size	Accuracy	No. genes with easy to design probes taken
50	87.16	31
75	88.69	43
100	88.64	60
125	88.22	76
150	88.34	93
175	88.66	110
200	88.57	131
225	89.28	151
250	88.72	172
275	88.43	195
300	88.40	212
325	88.19	240
350	87.69	258
375	87.31	286
400	86.80	312
425	86.15	345
450	85.85	366
475	85.71	385
500	85.92	385

Table S10: Accuracy and numbers of genes with easy to design probes included in the panel by scGIST when genes with easy to design probes are assigned priority score 1 and $\beta = 0.2$

Beta (β)	Accuracy	No. genes with easy to design probes taken
0	88.93	114
0.025	88.78	135
0.05	89.17	161
0.075	89.31	187
0.1	88.84	220
0.125	87.87	244
0.15	87.21	266
0.175	87.43	289
0.2	86.57	315
0.225	85.74	330
0.25	85.39	348
0.275	84.81	357
0.3	84.96	362
0.325	83.73	373
0.35	83.61	376
0.375	83.43	382
0.4	82.84	385
0.425	82.59	385
0.45	82.72	385
0.475	83.04	385
0.5	82.65	385

Table S11: Accuracy and numbers of genes with easy to design probes included in the panel by scGIST for varying β when genes with easy to design probes are assigned priority score 1 and panel size is 400

3 Supplementary Notes

3.1 Alternate regularization penalty for panel size

In the default configuration, scGIST is strict about panel size, which means the regularization function penalizes for including more or less genes than the given panel size. It may not always be the desired outcome. Sometimes we want to create a panel with as few genes as possible without sacrificing accuracy. In the alternate regularization function, we change the second part of the regularization function to $\alpha \max(||w||_1 - d, 0)$ from $\alpha|||w||_1 - d|$. Supplementary Figure S10 shows the regularization loss against the number of genes included in the panel for default and alternate regularization functions. The accuracy and the numbers of genes included in the panel for varying target panel sizes for the two modes are shown in Supplementary Figure S11.

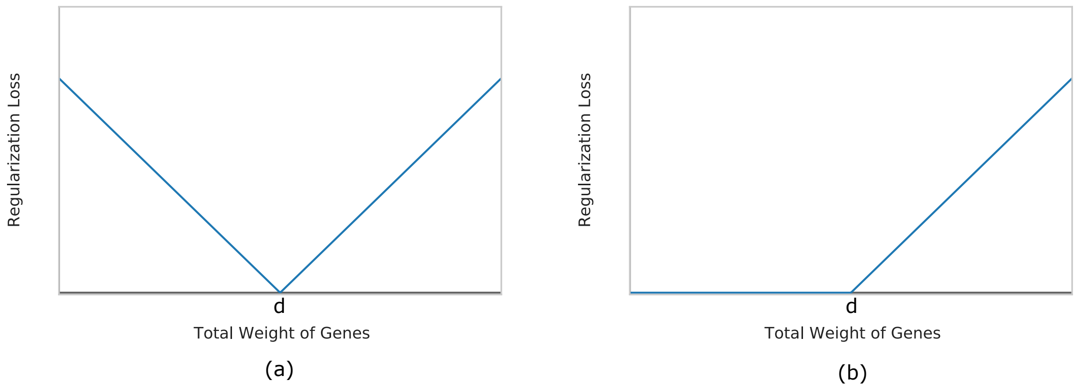

Figure S10: Comparison of regularization loss vs number of genes included in panels for (a) default and (b) alternate regularization functions.

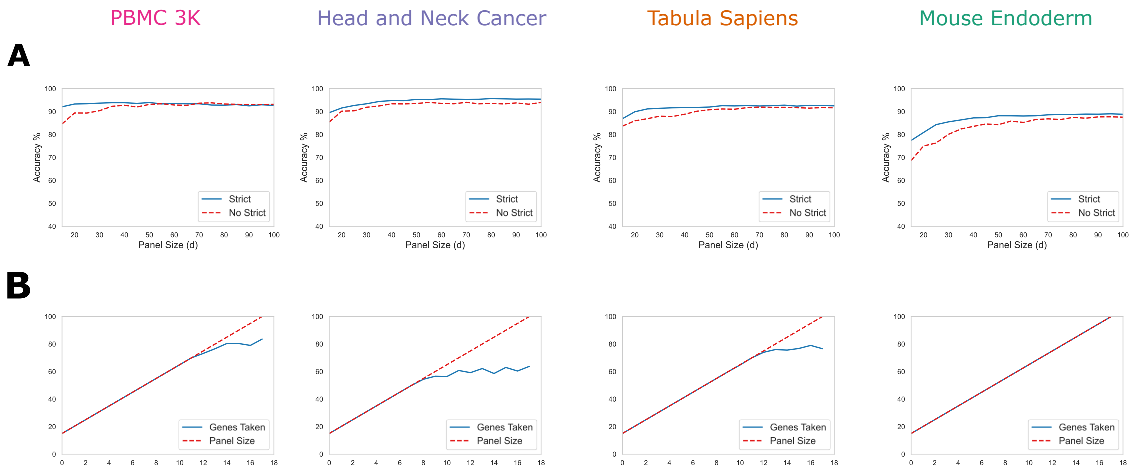

Figure S11: **A.** Accuracy of the default (strict) mode and the alternate (non-strict) mode for different datasets for varying target panel size, **B.** Comparison of target panel size and the numbers of genes included in the panel in for the two modes.

3.2 Evaluation metrics

Cell type prediction is multi-class classification problem with possible class imbalance. We used accuracy and macro F1-score as the evaluation metrics to assess various tools. Consider, a dataset with N samples and K possibles classes. Let, TP_k, TN_k, TP_k, TN_k be the number of true positives, true negatives, false positives and false negatives for the k-the class type. Then the accuracy is given by

$$Accuracy = \frac{\sum_{k=1}^{K} TP_k}{N}$$

However, accuracy can be misleading when there is class imbalance. So, we also use macro F1-score for assessment.

Precision and Recall for the k-th class type are given by

$$Precision_k = \frac{TP_k}{TP_k + FP_k}$$

$$Recall_k = \frac{TP_k}{TP_k + FN_k}$$

The macro average precision and recall are the averages over the precisions and recalls.

$$MacroAveragePrecision = \frac{\sum_{k=1}^{K} Precision_k}{K}$$

$$MacroAverageRecall = \frac{\sum_{k=1}^{K} Recall_k}{K}$$

The macro F1-score is then given by

$$MacroF1 - Score = 2\left(\frac{MacroAveragePrecision * MacroAverageRecall}{MacroAveragePrecision^{-1} + MacroAverageRecall^{-1}}\right)$$

3.3 Accuracies and Macro F1-Scores from Multiple Runs

Our analysis involved running the model five times and taking their average for each of the 4 datasets. Remarkably, the obtained results show minimal variability in both accuracy and macro F1-score. Figure S12 demonstrates the consistent results across multiple runs. This uniformity reinforces the stability and reliability of the deep learning model of scGIST.

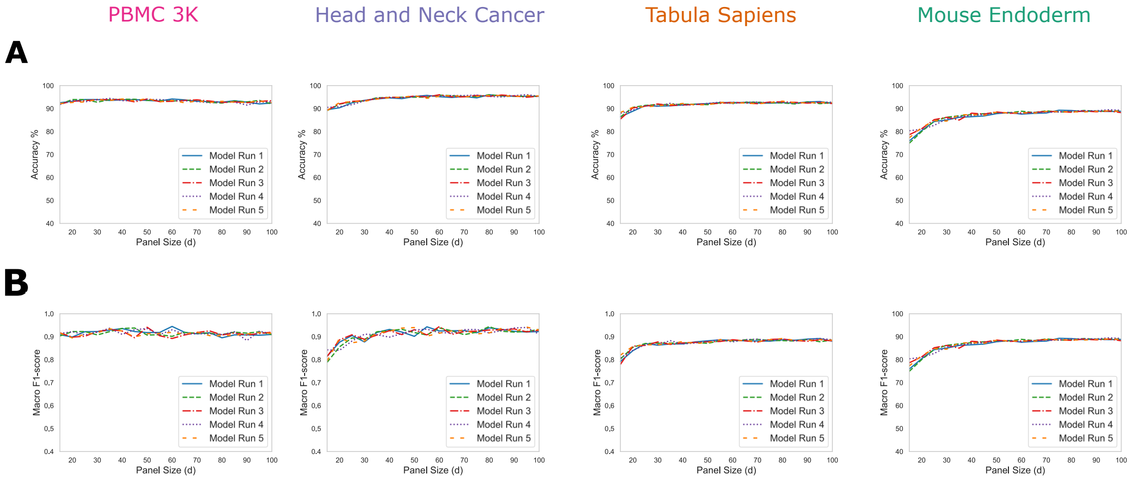

Figure S12: **A.** Accuracies and **B.** Macro F1-Scores of scGIST across multiple runs.
