# Supplementary material for: scGIST: gene panel design for spatial transcriptomics with prioritized gene sets
Source: Genome Biol. 2024 Feb 26;25:57. doi: 10.1186/s13059-024-03185-y (PMC10895727; doi:10.1186/s13059-024-03185-y)
Supplement: Supplementary file 2 — Additional file 2. Peer review history. [file 13059_2024_3185_MOESM2_ESM.docx]

**Review history**

**First round of review**

**Reviewer 1**

Yafi et al. present scGIST (single cell Gene-panel Inference for Spatial Transcriptomics), a deep learning-based method for selecting gene panels for FISH-based spatial transcriptomics. The input single-cell sequencing data is processed through a one-to-one feature selection layer, followed by three fully connected layers and then the output layer corresponding to the cell types. The loss function constrains the weights in the one-to-one layer so that each feature (gene) either has a weight of 0 or 1, corresponding to whether it is selected or not, and so that the total number of genes in the panel is close to a target number.

Compared to previous methods (scGeneFit, SMaSH, geneBasis), gene panels selected by scGIST demonstrate higher cell type prediction accuracy as well as Macro F1 score. In addition, scGIST is able to preferentially incorporate user-selected genes in the panel -- without significantly compromising the cell type prediction accuracy -- by penalizing the exclusion of these genes in the loss function. Furthermore, scGIST is able to preferentially include pairs of genes (for example, receptors / ligands) in the panel.

This paper presents a new approach for selecting gene panels for FISH-based spatial transcriptomic measurements, and demonstrates advances compared to existing methods. Therefore, I think this paper is worthy of publication. However, there are some minor concerns related to the methodology and interpretation that should be addressed.

\###### Concerns related to methodology and interpretation #####

Major concerns:

One major concern I have is on the differences between single-cell sequencing (sc-seq) and FISH-based spatial transcriptomic datasets, and whether gene panels derived from single-cell sequencing can be fully applicable to spatial transcriptomic measurements. Due to technical constraints, these two methods usually differ in the detection efficiency of individual genes, and the transcript composition of the same cell type can be very different between sc-seq and FISH-based ST methods. In this paper, for the selected gene panels, the authors measure their cell type prediction accuracy based on gene compositions from sc-seq datasets. It is understandable why the authors do so, as generating their own multiplexed FISH data would be a substantial challenge. Nonetheless, there should be a discussion on this point, and the authors should highlight where they think the limitations would be in the real world application of their panels.

Minor concerns:

In the abstract and introduction, the authors state that FISH-based ST can accommodate gene panel sizes of 10 ~ 250. However, this is no longer the case: commercial FISH-based ST solutions, offered by companies like Vizgen and NanoString, can do up to 500 ~ 1000 genes; In the academic setting, up to 10,000 genes have been measured at once. The authors should update their references to highlight recent papers that have characterized thousands of genes with these methods and should better discuss the state-of-the-art in their introduction.

The authors demonstrate that scGIST can preferentially include or exclude pairs of genes, such as receptor - ligand pairs. I wonder if scGIST can be extended to preferentially include not just pairs of genes, but a group of more than two genes. This can be useful in many contexts, such as receptor - ligand interactions that are not one-to-one, or multiple genes from the same complex or pathway.

The authors demonstrated the improved performance of scGIST over scGeneFit, geneBasis, and SMaSH and provide a very brief description of these previous methods. However, they provide no explanation of why their approach leads to improved performance and simply state that this is difficult to explain. A qualitative discussion on this point could help the reader understand the key intellectual advance(s) of scGIST.

Finally, we have some concerns related to the usefulness of the option to preferentially include (but not always include) a select subset of genes with the priority scores. First, the authors should define what they mean by "easy to design" probes. There are now a variety of FISH probe design packages that can design FISH probes for the vast majority of the transcriptome, so it is unclear why some genes should be given preference because they are easier to design probes for. Similarly, the ability to add in all genes within a given pathway is powerful. However, how should the reader interpret a panel in which only a subset of genes in the pathway are ultimately included within the panel?


\###### Concerns related to data presentation #####

Figure 1 F. This panel is missing a color bar.

Figure 2 D: In iii and iv, the colors are not distinct enough for the readers to distinguish between different cell types

**Reviewer 2**

- Are the methods appropriate to the aims of the study, are they well described, and are necessary controls included?
A deep learning based optimization framework was designed using RNA seq datasets from 4 different databases.
The idea about user defined genes to study custom pathways is interesting.

- Are the conclusions adequately supported by the data shown?
The selection of genes and expression of user defined ligand-receptor pairs are well demonstrated.

- Are sufficient details provided to allow replication and comparison with related analyses that may have been performed?
Yes and no. the RNA seq was properly analyzed, but the spatial transcriptomics data sets have not been used to validate the proposed method.
The replication of the same analysis multiple times should be also demonstrated using more replicates. Would the accuracies be different, if deep learning is tested at least 3 times etc.

- Is the method likely to be of broad utility? Is any software component easy to install and use? Please indicate briefly the novel features and/or advantages of the method, and/or please reference the relevant publications and which methods, if any, it should be compared with.
Use defined gene selection and deep learning based regulation framework will be interesting for the spatial transcriptomics community.

- Is the paper of broad interest to others in the field, or of outstanding interest to a broad audience of biologists?
Yes, the scGIST will be broadly applicable to many applications in biology.

Technically interesting and well developed method, but the final validations using spatial transcriptomics datasets would be crucial.
Confirming CellPhoneDB/CellChat spatially and making sure the best genes are selected and they will work the best for the biological finding would be exciting.

**Authors’ response to reviewers**

# Response to comments from Reviewer 1

*“Yafi et al. present scGIST (single cell Gene-panel Inference for Spatial Transcriptomics), a deep learning-based method for selecting gene panels for FISH-based spatial transcriptomics. The input single-cell sequencing data is processed through a one-to-one feature selection layer, followed by three fully connected layers and then the output layer corresponding to the cell types. The loss function constrains the weights in the one-to-one layer so that each feature (gene) either has a weight of 0 or 1, corresponding to whether it is selected or not, and so that the total number of genes in the panel is close to a target number.*

*Compared to previous methods (scGeneFit, SMaSH, geneBasis), gene panels selected by scGIST demonstrate higher cell type prediction accuracy as well as Macro F1 score. In addition, scGIST is able to preferentially incorporate user-selected genes in the panel -- without significantly compromising the cell type prediction accuracy -- by penalizing the exclusion of these genes in the loss function. Furthermore, scGIST is able to preferentially include pairs of genes (for example, receptors / ligands) in the panel.*

*This paper presents a new approach for selecting gene panels for FISH-based spatial transcriptomic measurements, and demonstrates advances compared to existing methods. Therefore, I think this paper is worthy of publication. However, there are some minor concerns related to the methodology and interpretation that should be addressed.”*

We thank the reviewer for their positive comments about our work and overall finding it “*worthy of publication*”. We also appreciate the reviewer’s suggestions. As we discuss in our

point-by-point response below, we have revised our text, figures, and added three new analyses to incorporate those suggestions. We hope the reviewer finds these revisions satisfactory.

*“One major concern I have is on the differences between single-cell sequencing (sc-seq) and FISH-based spatial transcriptomic datasets, and whether gene panels derived from single-cell sequencing can be fully applicable to spatial transcriptomic measurements. Due to technical constraints, these two methods usually differ in the detection efficiency of individual genes, and the transcript composition of the same cell type can be very different between sc-seq and FISH-based ST methods. In this paper, for the selected gene panels, the authors measure their cell type prediction accuracy based on gene compositions from sc-seq datasets. It is understandable why the authors do so, as generating their own multiplexed FISH data would be a substantial challenge. Nonetheless, there should be a discussion on this point, and the authors should highlight where they think the limitations would be in the real world application of their panels.”*

We thank the reviewer for mentioning this critical point. We have now added a detailed discussion (4th paragraph in Discussion), summarizing the following points:

One potential limitation of scGIST is, similarly to other tools for gene panel design, it builds gene panels using single-cell/nucleus (sc/sn) RNA-seq data. The panels will be of limited use if the two technologies differ substantially in their transcript detection efficiency. However, recent literature suggests that the two technologies generally agree in transcript detection and quantification. Correlations between MERFISH/Vizgen/SeqFISH+ data and RNA-seq FPKM values were shown to be as high as 0.94 and are generally around 0.8.

Although two studies reported correlations of 0.5 and 0.6, this is largely due to a drop in correlations for genes expressed at low levels. Therefore, to test if scGIST is biased towards genes with low transcript counts, we analyze its performance excluding the genes with expressions in the lowest ten percentile. The results (Supplementary Figure S9) show that the accuracy of scGIST does not decrease substantially even if we exclude the genes with very low expression levels.

Overall, we expect that the gene panels designed based on the expression levels from scRNA-seq datasets will be able to successfully distinguish different cell types in spatial transcriptomics experiments. To ensure scGIST’s applicability, we recommend excluding or de-prioritizing genes with very low transcript counts.

*“In the abstract and introduction, the authors state that FISH-based ST can accommodate gene panel sizes of 10 ~ 250. However, this is no longer the case: commercial FISH-based ST solutions, offered by companies like Vizgen and NanoString, can do up to 500 ~ 1000 genes; In the academic setting, up to 10,000 genes have been measured at once. The authors should update their references to highlight recent papers that have characterized thousands of genes with these methods and should better discuss the state-of-the-art in their introduction.”*

We thank the reviewer for this suggestion. We have now added more recent references (References 13-15) and cross-checked our compiled bibliography with that maintained at the Museum of Spatial Transcriptomics (Lior Pachter's live resource accompanying their 2022 Nature Methods article). While the majority of techniques allow panels with a few hundred genes, the commercially available technologies Vizgen and Nanostring offer panels of 500 to

~1000 genes (<https://www.nature.com/articles/s41587-022-01483-z>). However, in studies where one wishes elaborate cataloging of cell states (or cell subtypes), panel sizes will remain a bottleneck requiring the use of tools like ours and others.

The reviewer is also correct that SeqFISH+, an academic (non-commercial) technology, has targeted a panel of ~10,000 genes in the mouse olfactory bulb. With such potential to scale up, SeqFISH+ will indeed be a game changer if it starts demonstrating the ability to target other cell- and context-specific genes. It will also be necessary that SeqFISH+ is made broadly accessible within affordable budgets, although the point on affordability is still unclear. As such, we anticipate that tools like scGIST will continue serving the community until we have a truly scalable and accurate sc-ST method.

*“The authors demonstrate that scGIST can preferentially include or exclude pairs of genes, such as receptor - ligand pairs. I wonder if scGIST can be extended to preferentially include not just pairs of genes, but a group of more than two genes. This can be useful in many contexts, such as receptor - ligand interactions that are not one-to-one, or multiple genes from the same complex or pathway.”*

We thank the reviewer for raising this point. scGIST can indeed be extended to preferentially include complexes with more than two genes. As indicated in the “Inclusion of complexes of

interest (receptor-ligand complexes)” section, we encode a complex with *n* genes using (𝑛)

2

pairs. All these pairs are then added to the regularization function thus penalizing exclusion of any of the genes in the complex. We have expanded the section “Inclusion of complexes of interest“ to discuss multi-gene complexes and added Supplementary Figures S7 and S8.

*“The authors demonstrated the improved performance of scGIST over scGeneFit, geneBasis, and SMaSH and provide a very brief description of these previous methods. However, they provide no explanation of why their approach leads to improved performance and simply state that this is difficult to explain. A qualitative discussion on this point could help the reader understand the key intellectual advance(s) of scGIST.”*

We have elaborated on the probable reasons scGIST outperforms other methods in the Discussion section (the 3rd paragraph). One of the strengths of scGist over other methods is it explicitly incorporates the panel size as a constraint in the regularization function. SMaSH learns a classifier without taking panel size into account and then selects the top genes into the panel. geneBasis greedily takes the top genes into the panel i.e. at each step takes the gene which leads to the most improvement in results as long as the panel is not full. scGeneFit does consider panel size, but it relies on linear programming. In contrast, our method uses a

non-linear neural network, enabling us to explore a larger space of functions.

*“Finally, we have some concerns related to the usefulness of the option to preferentially include (but not always include) a select subset of genes with the priority scores. First, the authors should define what they mean by "easy to design" probes. There are now a variety of FISH probe design packages that can design FISH probes for the vast majority of the transcriptome, so it is unclear why some genes should be given preference because they are easier to design probes for. Similarly, the ability to add in all genes within a given pathway is powerful. However, how should the reader interpret a panel in which only a subset of genes in the pathway are ultimately included within the panel?”*

We apologize for this confusing term. We wanted to refer to *easy-to-target genes*. We have now clarified the reasons that make it difficult for FISH-based technologies to target a considerable number of genes in the section “Prioritization of easy-to-target genes”. These challenges, combined with the need for error-correcting barcodes, have so far limited the number of targetable genes to ~10,000 (half of the transcriptome). First, FISH-based methods use probes to target transcripts. The fluorescent barcodes attach to these probes. For robust quantification,

there needs to be a sufficient number of targetable regions per transcript. Second, targeting specific transcripts becomes challenging for genes with high sequence similarity, e.g., genes in the human olfactory receptor family. Finally, highly expressed genes – because of their large number of transcripts – create optical crowding in the FISH images, making it difficult to target those genes.

The reviewer has raised an important point about how a user should interpret a panel in which only a subset of genes from a targeted pathway was included. We have now clarified that in those circumstances, a user needs to adopt an iterative approach by re-weighting the genes in their desired pathway. At its core, panel design is a combinatorial optimization problem and scGIST facilitates assessing the extent to which a user can best utilize a size-constrained gene panel.

First, by iterative applications of the algorithm – every time increasing the weights of genes in the desired pathway through the priority coefficient (□) – a user can assess the tradeoff between classification accuracy and the number of desired genes that can be included in the panel.

Second, even when all of the desired genes cannot be accommodated, a user can iteratively apply scGIST to identify if different meaningful subsets of the desired genes can be selected. Third, a user can also use scGIST to systematically estimate an increased panel size necessary to accommodate a satisfactory number of their desired genes. Based on these outcomes, a user can then decide to proceed with a subset of the genes or increase the panel size. We wish to note that the commercial Vizgen pipeline indeed applies an iterative approach for designing panels, albeit scGIST offers a more versatile use-case.

We have expanded the section “Inclusion of other genes of interest” and added a figure in the supplementary (Supplementary Figure S6) to show how priority coefficient (□) can be increased to include all the YAP-target genes without compromising accuracy greatly.

*“Figure 1 F. This panel is missing a color bar.”*

We have added the color bar.

*“Figure 2 D: In iii and iv, the colors are not distinct enough for the readers to distinguish between different cell types”*

We thank the reviewer for the observation. We have changed our color palette to make different cell types more easily distinguishable.

# Response to comments from Reviewer 2

*“- Are the methods appropriate to the aims of the study, are they well described, and are necessary controls included?*

*A deep learning based optimization framework was designed using RNA seq datasets from 4 different databases.*

*The idea about user defined genes to study custom pathways is interesting.*

*- Are the conclusions adequately supported by the data shown?*

*The selection of genes and expression of user defined ligand-receptor pairs are well demonstrated.”*

We are pleased that the reviewer found the idea of prioritizing genes for inclusion in the panel to study specific pathways interesting, and the experimental validations convincing.

*“- Are sufficient details provided to allow replication and comparison with related analyses that may have been performed?*

*Yes and no. the RNA seq was properly analyzed, but the spatial transcriptomics data sets have not been used to validate the proposed method.”*

We thank the reviewer for the comments. We had to resort to validating our method with scRNA-seq data, as done by previous methods, as spatial transcriptomics data sets with gene expressions for a large number of genes and cell type labels were not available and it was beyond our scope to generate new ones.

The panels will be of limited use if the two technologies differ substantially in their transcript detection efficiency. However, recent literature suggests that the two technologies generally agree in transcript detection and quantification. Correlations between MERFISH/Vizgen/SeqFISH+ data and RNA-seq FPKM values were shown to be as high as

0.94 and are generally around 0.8. Although two studies reported correlations of 0.5 and 0.6, this is largely due to genes with low expressions. We have shown that even if we exclude genes with expressions in the lowest ten percentile, the accuracy of scGIST does not decrease substantially (Supplementary Figure S9).

*“The replication of the same analysis multiple times should be also demonstrated using more replicates. Would the accuracies be different, if deep learning is tested at least 3 times etc.”*

The accuracies and macro F1 scores we report for scGIST are actually averages over 5 runs. We have added Supplementary Note 3.3 that shows values for each run. We observe that the accuracies and macro F1 scores do not vary substantially across runs demonstrating the consistency in performance of scGIST.

*“- Is the method likely to be of broad utility? Is any software component easy to install and use? Please indicate briefly the novel features and/or advantages of the method, and/or please reference the relevant publications and which methods, if any, it should be compared with.*

*Use defined gene selection and deep learning based regulation framework will be interesting for the spatial transcriptomics community.*

*- Is the paper of broad interest to others in the field, or of outstanding interest to a broad audience of biologists?*

*Yes, the scGIST will be broadly applicable to many applications in biology.*

*Technically interesting and well developed method, but the final validations using spatial transcriptomics datasets would be crucial.*

*Confirming CellPhoneDB/CellChat spatially and making sure the best genes are selected and they will work the best for the biological finding would be exciting.”*

We are delighted that the reviewer found the method interesting and well developed and concurs with us that the method will be broadly applicable. We validated our method using scRNA-seq datasets, and based on the observed correlations in gene expression estimates between scRNA-seq and spatial transcriptomics technologies, we believe that the gene panels designed by scGIST will be able to distinguish among different cell types.

We demonstrated how scGIST can be used to include complexes and genes from specific pathways in the gene panels and provided examples of insights that can be derived from such panels. Like the reviewer, we are excited about the types of novel analysis scGIST will enable and the biological findings that will lead to.

**Second round of review**

**Reviewer 1**

We thank the authors for going through our point-by-point concerns, and addressing each one of them. We believe the paper is now suitable for publication.

Nevertheless, we believe that this paper can be further strengthened if the authors can revise their responses to two of our comments:

------------------------------------------------------------------------------------------------------------------------------
"One potential limitation of scGIST is, similarly to other tools for gene panel design, it builds gene panels using single-cell/nucleus (sc/sn) RNA-seq data. The panels will be of limited use if the two technologies differ substantially in their transcript detection efficiency. However, recent literature suggests that the two technologies generally agree in transcript detection and quantification. Correlations between MERFISH/Vizgen/SeqFISH+ data and RNA-seq FPKM values were shown to be as high as 0.94 and are generally around 0.8.
Overall, we expect that the gene panels designed based on the expression levels from scRNA-seq datasets will be able to successfully distinguish different cell types in spatial transcriptomics experiments. To ensure scGIST's applicability, we recommend excluding or de-prioritizing genes with very low transcript counts."

For this response, we do not fully agree with the statement that the good correlation between imaging-based transcriptomic methods and bulk RNA-seq is a good indication that single-cell RNA-seq datasets will be informative for designing imaging-based transcriptomic panels, especially in the cell-type resolved context. Bulk and single-cell RNA-seq are two very different methods with differential sensitivities to different cell types and genes. Furthermore, imaging-based single-cell spatial transcriptomic methods have been proven able to discover cell types not present in single-cell datasets, due to the ability of these methods to have higher throughput (in terms of the number of cells) and higher sensitivity to the targeted genes.

Perhaps the authors can acknowledge that gene panels designed from single-cell studies, while informative, cannot be perfect or comprehensive for imaging-based assays. The users can use these gene panel outputs as a starting point, but some manual checking combined with domain expertise is always required.
------------------------------------------------------------------------------------------------------------------------------
"The reviewer is also correct that SeqFISH+, an academic (non-commercial) technology, has targeted a panel of ~10,000 genes in the mouse olfactory bulb. With such potential to scale up, SeqFISH+ will indeed be a game changer if it starts demonstrating the ability to target other cell- and context-specific genes. It will also be necessary that SeqFISH+ is made broadly accessible within affordable budgets, although the point on affordability is still unclear. As such, we anticipate that tools like scGIST will continue serving the community until we have a truly scalable and accurate sc-ST method."

For this response, we recommend that the authors do a more comprehensive literature research on the most recent FISH-based spatial transcriptomic studies. The SeqFISH+ paper is not the only academic study with 10,000+ genes, and there are other studies with thousands of genes. A better review of the literature can give the readers a more objective view of the landscape of the field.
------------------------------------------------------------------------------------------------------------------------------

**Reviewer 2**

Authors have addressed majority of questions. Two minor concerns:
1) Benchmarking of selected genes in spatial transcriptomics/MERFISH/seqFISH or similar data sets are still lacking. Spatial validation of priority genes would be convincing for readers
2) Figure axis labels are too small to read. Authors should increase the font size of each labels (eg., Fig 2C) and keep consistent throughout the entire manuscript.
3) Authors should prepare a read me file and present visual examples and test dataset in their GitHub

**Authors’ response to reviewers**

In the current version, we have incorporated all the suggestions from you and the reviewers; a summary is as follows. 1. Following the suggestion of Reviewer 2, we now showed a new analysis where we benchmark scGIST and geneBasis on a seqFISH+ dataset. A new subsection “Evaluation on a spatial transcriptomics dataset” has been added in “Results” on page 8 to describe the experiment and the findings are shown in Figure 4. 2. Following the suggestion of Reviewer 1, we now updated our introductory review of the literature on currently available single-cell resolution ST technologies. We now refer to the recent commercially available technologies from Vizgen, Rebus, and Nanostring, as well as seqFISH+ and RNA SPOTs, the academic approaches that have shown the ability to use about 10,000 genes. 3. We have incorporated all editorial suggestions regarding formatting, data and code deposition, licensing, and rewriting the Abstract within a 100-word limit. We have also provided the figures in vector graphics format (.eps), which will resolve the readability issues.
